# Untargeted and Targeted Metabolomics Reveal the Active Peptide of *Eupolyphaga sinensis* Walker against Hyperlipidemia by Modulating Imbalance in Amino Acid Metabolism

**DOI:** 10.3390/molecules28207049

**Published:** 2023-10-12

**Authors:** Yanan Li, Pingping Dong, Long Dai, Shaoping Wang

**Affiliations:** 1 School of Pharmacy, Shandong University of Traditional Chinese Medicine, Jinan 250300, China; 18353199305@163.com; 2 School of Pharmacy, Binzhou Medical University, Yantai 264003, China; 17864191173@163.com; 3 State Key Laboratory for Quality Research of Chinese Medicines, Macau University of Science and Technology, Avenida Wai Long, Macao SAR 999078, China

**Keywords:** active peptide (APE) of *Eupolyphaga sinensis* Walker, hyperlipidemic, untargeted and targeted metabolomics, amino acid metabolism

## Abstract

The active peptide (APE) of *Eupolyphaga sinensis* Walker, which is prepared by bioenzymatic digestion, has significant antihyperlipidemic effects in vivo, but its mechanism of action on hyperlipidemia is not clear. Recent studies on amino acid metabolism suggested a possible link between it and hyperlipidemia. In this study, we first characterized the composition of APE using various methods. Then, the therapeutic effects of APE on hyperlipidemic rats were evaluated, including lipid levels, the inflammatory response, and oxidative stress. Finally, the metabolism-regulating mechanisms of APE on hyperlipidemic rats were analyzed using untargeted and targeted metabolomic approaches. The results showed that APE significantly reduced the accumulation of fat, oxidative stress levels, and serum pro-inflammatory cytokine levels. Untargeted metabolomic analysis showed that the mechanism of the hypolipidemic effect of APE was mainly related to tryptophan metabolism, phenylalanine metabolism, arginine biosynthesis, and purine metabolism. Amino-acid-targeted metabolomic analysis showed that significant differences in the levels of eight amino acids occurred after APE treatment. Among them, the expression of tryptophan, alanine, glutamate, threonine, valine, and phenylalanine was upregulated, and that of arginine and proline was downregulated in APE-treated rats. In addition, APE significantly downregulated the mRNA expression of SREBP-1, SREBP-2, and HMGCR. Taking these points together, we hypothesize that APE ameliorates hyperlipidemia by modulating amino acid metabolism in the metabolome of the serum and feces, mediating the SREBP/HMGCR signaling pathway, and reducing oxidative stress and inflammation levels.

## 1. Introduction

*Eupolyphaga sinensis* Walker (*E. sinensis*) has been consumed in East Asia because of its protein, amino acids, unsaturated fatty acids, and trace elements [1,2,3]. Previous studies have confirmed the many biological activities of *E. sinensis*, including its antioxidant, antithrombotic, and anti-inflammatory effects due to its high protein content. Moreover, *E. sinensis* has been used as a traditional Chinese medicine for the treatment of hyperlipidemia for 2000 years [4], and current studies have confirmed similar functions. It is well known that after oral administration, proteins cannot be directly absorbed by the human circulatory system due to their large molecular weight and complex structure, and they can only be digested into bioactive peptides by pepsin, trypsin, and other digestive enzymes. Compared with proteins, bioactive peptides have several advantages, including low molecular weight, easy absorption, and low energy consumption [5,6]. As a result, bioactive peptides exhibit greater bioactivity than the precursor proteins.

Hyperlipidemia (HL) is a common symptom of metabolic disorders and is closely associated with Type 2 diabetes, obesity, insulin resistance, DNA damage, and non-alcoholic fatty liver disease [7,8,9]. It often manifests as an abnormal increase in total cholesterol (TC), triglycerides (TG), and low-density lipoprotein cholesterol (LDL-C), and a decrease in high-density lipoprotein cholesterol (HDL-C). Despite significant advances in pharmacological treatment, approximately 1.5 billion people in the world still suffer from HL. Currently, the most widely used lipid-lowering drugs in clinical practice include statins, fibrates, and niacin [10,11]. However, the long-term use of these drugs may lead to serious adverse effects. Therefore, the development of new safe and effective antihyperlipidemic drugs is of great importance. Bioactive peptides derived from animal-based Chinese medicine are considered to be potential substitutes for the prevention and treatment of HL due to their good potency and low toxicity [12].

Metabolomic analysis, as a powerful qualitative and quantitative tool, has been widely used to explore the endogenous metabolites of diseases. It can quantify the levels of metabolites and elucidate the mechanisms of action of diseases and drugs. There are two types of metabolomic approach: untargeted and targeted. Untargeted metabolomics is used for the characterization of metabolites and metabolic profiling analyses, and involves more abundant information. However, targeted metabolomics is mainly used for the quantification of metabolites, such as amino acids, bile acids, fatty acids, and other components, and involves more precise information [13]. Currently, metabolomic technologies are used in the fields of diagnosis, monitoring, and developing drugs for HL.

In this study, the active peptide of *E. sinensis* (APE) was obtained via enzymatic hydrolysis of *E. sinensis*. Firstly, an HL rat model induced by a high-fat diet was established to evaluate the hypolipidemic efficacy of APE. Then, an untargeted metabolomic analysis of the serum and feces was used to identify the key differential metabolites and metabolic pathways through which APE can ameliorate HL. A targeted metabolomic analysis of amino acids was also performed. Finally, the mRNA expression of genes related to lipid metabolism was detected via real-time quantitative PCR (RT-PCR) to preliminarily reveal the molecular mechanism of APE against HL. Therefore, the aim of the study was to evaluate the efficacy of APE against HL and to provide a theoretical basis for exploring potential biomarkers or therapeutic targets.

## 2. Results

### 2.1. Structural Elucidation of APE

**Figure 1:**
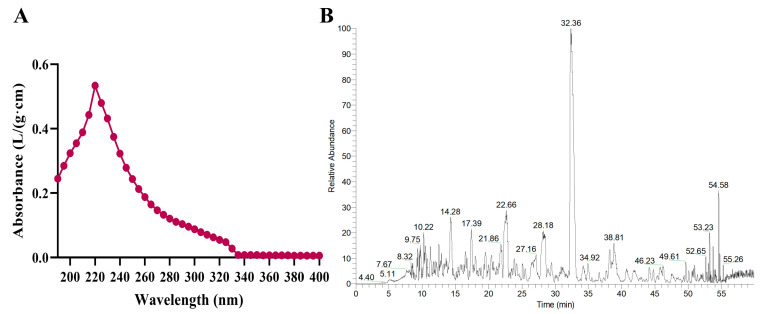
The results of APE molecular weight. (**A**) the results of a full-wavelength scan; (**B**) the base peak plot of the APE.

The peptide content in APE, as determined by the Folin phenol reagent method was 623.56 ± 17.10 mg/g. The sugar content in APE was 35.22 ± 0.72 mg/g (Table S1). This indicated that APE was probably a mixture that also contains inorganic salts due to the adjusted pH. As a result, 10 mg APE was dissolved in 100 mL deionized water, and the pH of APE was 8.12 ± 0.26 (*n* = 6) under these conditions (Table S2). APE was detected by scanning with a full UV wavelength of 190–400 nm, and the results showed that APE had the maximum absorption value at 220 nm. Therefore, 220 nm was set as the optimal absorption wavelength for APE in the next experiment (Figure 1A). Moreover, we used Easy nLC1200/Q Exactive Plus to identify and analyze the structure of APE. The results showed that the molecular weight of APE was concentrated between 90 Da and 1600 Da, and the peptides were mostly composed of 7–15 amino acids. The majority of the peptide’s C-terminal residues were lysine (K) and arginine (R). Figure 1B shows a diagram of the base peak of APE in the positive ion mode. Table S3 shows 60 peptides with high peak intensities.

### 2.2. Effects of APE on Blood Lipid Levels in HL Rats

**Figure 2:**
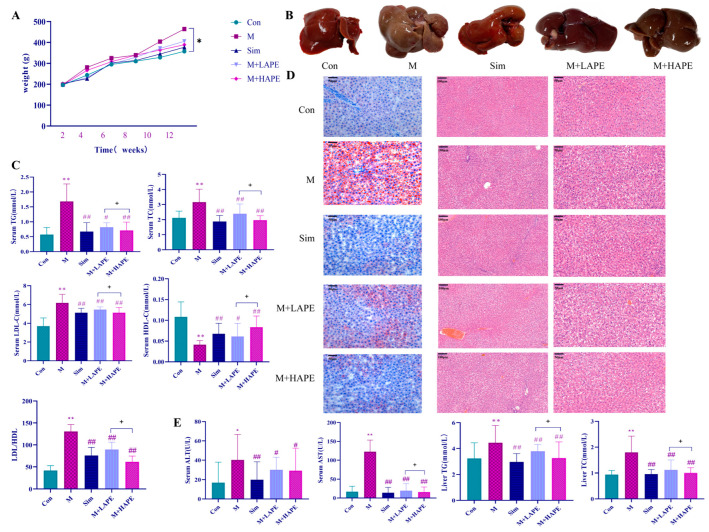
The results of anti-HL activity of APE. (**A**) Statistics of body weight change of rats in test groups; (**B**) Visual observation of rat liver in different groups; (**C**) TG, TC, LDL-C, and HDL-C levels in serum from all rats; (**D**) The results of Oil-Red O staining (**left**, 50 μm) and H&E staining (**right**, 100 μm and 50 μm); (**E**) AST, ALT, TG, and TC in serum and liver from all rats. n = 10, data are represented as means ± SD. * *p* < 0.05, ** *p* < 0.01, M group vs. Con group; # *p* < 0.05, ## *p* < 0.01, M group vs. treatment groups, + *p* < 0.05, M + LAPE vs. M + HAPE.

No animals died during the experiment. The body weights of the HFD-induced rats increased dramatically compared with the control group (Con) (*p* < 0.05). Different doses of APE reversed the weight gain in HFD-induced rats during the three weeks of treatment (Figure 2A). TG, TC, and LDL-C levels were significantly increased in the M group compared with the Con group (*p* < 0.01), while HDL-C levels were greatly decreased (*p* < 0.01), indicating that HFD induces HL in rats. Interestingly, different doses of APE reduced the levels of TC, TG, LDL-C, and HDL-C, and the LDL-C/HDL-C ratio was significantly higher (*p* < 0.01). In addition, the activity of high-dose APE was superior to that of low-dose APE, with a significant trend (*p* < 0.05). These findings suggested the therapeutic effect of APE on HL. The results are shown in Figure 2C.

### 2.3. Histopathologic Analysis

To determine the extent of cellular fibrosis and lipid accumulation, histopathological changes in the liver were studied by H&E staining and Oil Red O staining. The color of the liver of the M group showed a distinct yellow color compared with the Con group (Figure 2B). The pathological examination showed that the livers in the M group were filled with a large number of lipid droplets and a small number of inflammatory cells (neutrophils), indicating that HFD feeding had led to severe microvascular steatosis, hepatic steatosis, and increased lipid deposition. However, the morphology of the liver tissue and the accumulation of hepatic lipid were significantly improved by APE and the simvastatin treatment (*p* < 0.05), indicating that both APE and simvastatin treatment significantly reduced lipid deposition, and attenuated hepatic steatosis and liver injury (Figure 2D). These results indicated that the M + HAPE treatment showed significant efficacy against HL. As a result, the M + HAPE treatment was selected for the subsequent untargeted and targeted metabolomic test.

### 2.4. Effects of APE on Liver Injury in HL Rats

In order to evaluate the liver function of the rats, the levels of ALT and AST in the liver, as well as those of TG and TC, were determined. The results showed that the four parameters in the M group were obviously elevated compared with the Con group (*p* < 0.05). In the treated group, APE significantly reduced AST and ALT in the serum and TG and TC in the liver (*p* < 0.05, Figure 2E). Except for AST, the M + HAPE treatment controlled the above indices better than M + LAPE treatment.

### 2.5. Effects of APE on Oxidative Stress Levels and Inflammatory Factors in HL Rats

**Figure 3:**
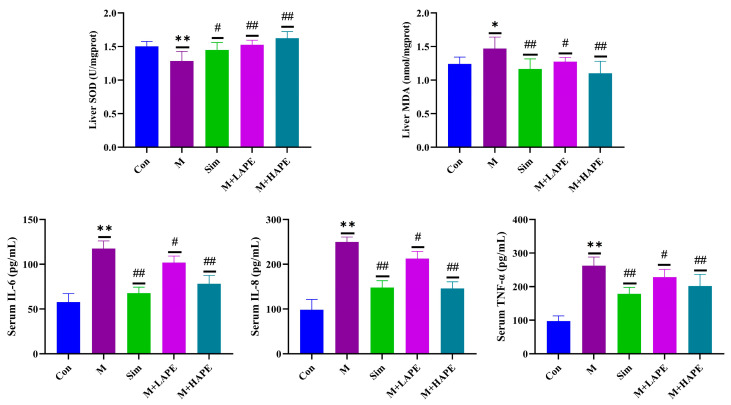
Changes in APE in liver SOD, liver MDA, serum IL-6, serum IL-8, and serum TNF-α levels in HL rats. * *p* < 0.05, ** *p* < 0.01, M group vs. Con group; # *p* < 0.05, ## *p* < 0.01, M group vs. treatment groups.

The effects of APE on oxidative stress in HL rats were evaluated by measuring the liver levels of SOD and MDA. Compared with the Con group, the liver levels of SOD were significantly lower (*p* < 0.01), and the levels of MDA were significantly higher (*p* < 0.01) in the M group. APE and simvastatin treatment significantly increased the liver levels of SOD (*p* < 0.01) and decreased the levels of MDA (*p* < 0.05) in the HL rats. The results suggest that a HFD could lead to oxidative stress in the liver. In addition, the levels of IL-6, IL-8, and TNF-α in the serum were measured via an ELISA to evaluate the effect of APE on the inflammatory response in HL rats. The results showed that serum levels of IL-6, IL-8, and TNF-α were significantly higher in the M group than in the Con group (*p* < 0.01). The APE and simvastatin intervention reversed this trend, and the M + HAPE group showed better effects than the M + LAPE group (Figure 3). These results illustrated that the development of HL is closely related to oxidative stress and the inflammatory response.

### 2.6. Serum and Feces Metabolic Profiling

#### Metabolic Profiling of Serum and Feces

PCA is an unsupervised pattern recognition analysis method that visualizes differences between samples in a multidimensional space. To explore the changes in the endogenous components in APE-treated HL rats, multivariate PCA and PLS-DA analyses were performed using SCIMA P_14.0 software. Figure S1 illustrates the base peak plots of the QC samples of the positive and negative ions for the serum and feces samples.

**Figure 4:**
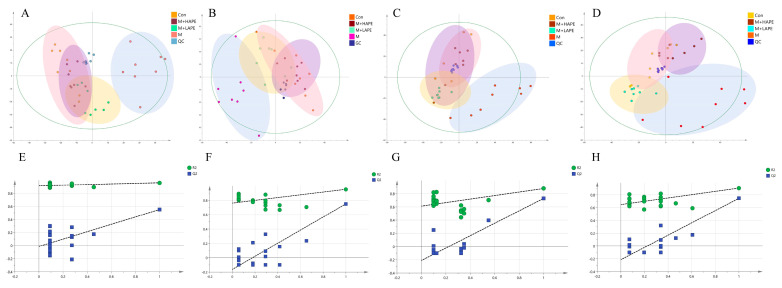
Results of a PCA analysis based on SCIMA_P 14.0. (**A**) PCA analysis of feces samples in negative ion mode; (**B**) PCA analysis of feces samples in positive ion mode; (**C**) PCA analysis of serum samples in negative ion mode; (**D**) PCA analysis of serum samples in positive ion mode; (**E**) PLS-DA replacement analysis results of feces samples in negative ion mode; (**F**) PLS-DA replacement analysis results of feces samples in positive ion mode; (**G**) PLS-DA replacement analysis results of serum samples in negative ion mode; (**H**) PLS-DA replacement analysis results of serum samples in positive ion mode. Con: control group, M: model group, M + LAPE: low-dose group of APE, M + HAPE: high-dose group of APE.

For the metabolomic analysis of the serum, the QC samples were tightly clustered together in the PCA score plot, indicating that the system was relatively stable during the analysis. As can be seen in Figure 4A,B, the points of the samples of the Con group were farther apart from those of the other groups, indicating that the serum metabolites of the rats in the M group were significantly altered compared with the Con group. The M group was not completely separate from the APE intervention group, and there was a certain degree of overlap, suggesting that the APE had a certain effect on HL. The points sampled from the M + LAPE and M + HAPE groups were clustered together and not separated, indicating that the metabolic differences in the serum between APE groups at different doses were not significant. In addition, PLS-DA achieved the maximum separation of the samples by building mathematical models for the different groups. In the positive and negative ion models, the values of R^2^Y of the PLS-DA models built for the M and M + HAPE groups were 0.992 and 0.937, and the values of Q^2^ were 0.703 and 0.984, respectively. The results of the test with 200 permutations showed that the PLS-DA model was not overfitted (Figure 4E,F). More importantly, the PLS-DA score plots revealed (Figure S2A,B) that the sample points were relatively well separated among the M, Con, M + LAPE, and M + HAPE groups, suggesting that there were differences in the serum metabolites between the APE and M groups. Therefore, the APE treatment was effective in lowering blood lipids and preventing HL.

For the metabolomic analysis of feces, the QC samples were tightly clustered. Similar to the results for the metabolomics of the serum, both the PCA score plot (Figure 4C,D) and the PLS-DA score plot (Figure S2C,D) revealed that there was a tendency for the Con, M, and M + HAPE groups to separate in the positive and negative ion modes. The APE treatment group was closer to the Con group, and was significantly separated from the M group, suggesting that APE modulated the metabolites in the feces of HFD-induced HL rats. In the PLS-DA analysis, the values of R^2^Y (1.0 and 0.983) and Q^2^ (0.875 and 0.711) in the negative and positive ion modes also showed that the model had better discriminatory and predictive ability (Figure 4G,H).

### 2.7. Screening and Identification of Differentially Expressed Metabolites

**Figure 5:**
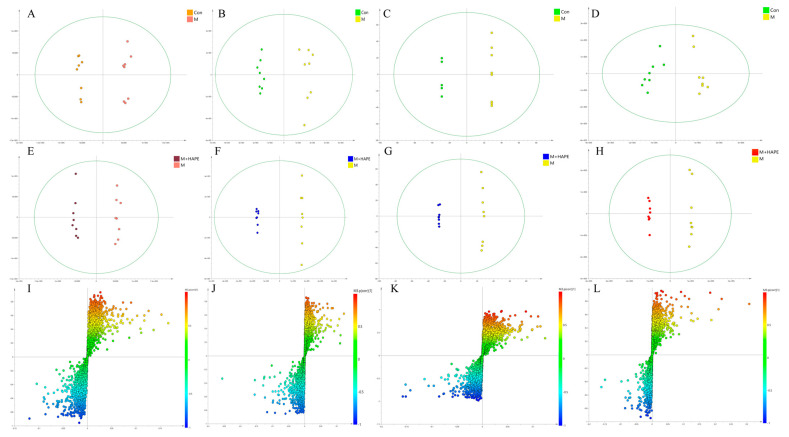

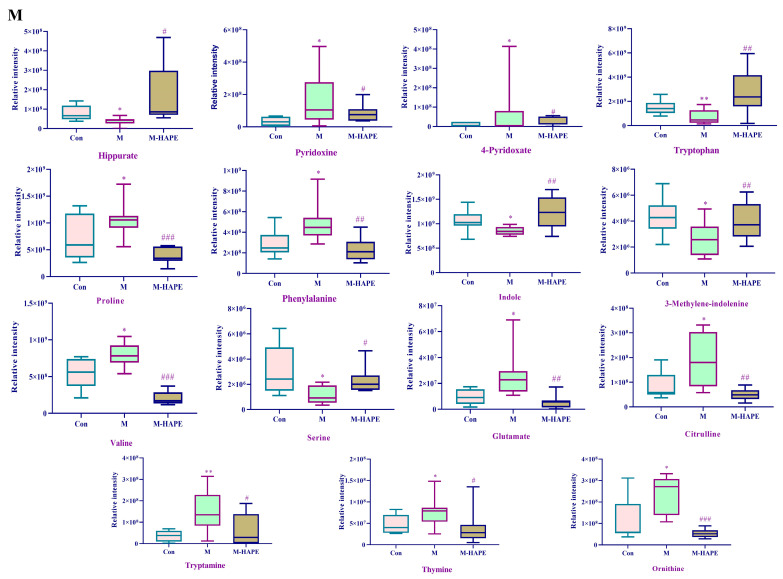

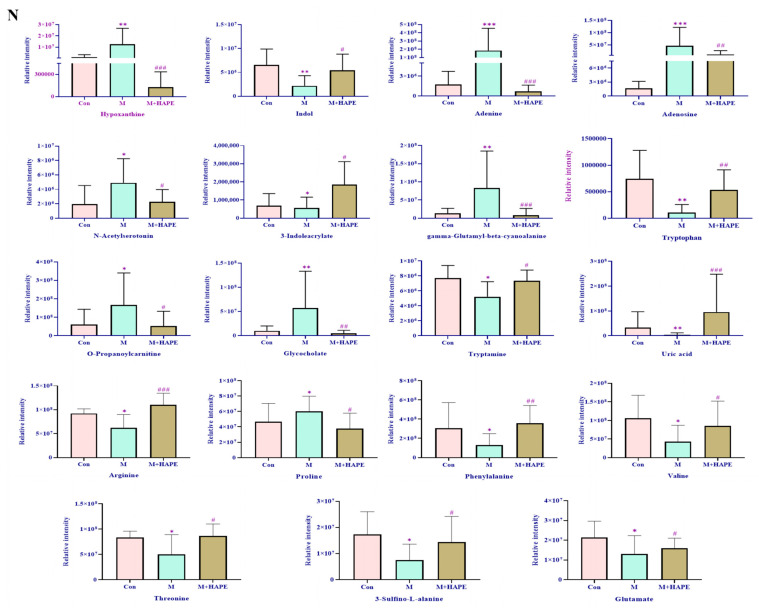
Results of OPLS-DA analysis and S-plot scores based on SCIMA_P 14.0. (**A**,**E**) OPLS-DA results of feces samples in negative ion mode ((**A**): M group vs. Con group, (**E**): M group vs. M + HAPE group); (**B**,**F**) OPLS-DA results of feces samples in positive ion mode ((**B**): M group vs. Con group, (**F**): M group vs. M + HAPE group); (**C**,**G**) OPLS-DA results of serum samples in negative ion mode ((**C**): M group vs. Con group, (**G**): M group vs. M + HAPE group); (**D**,**H**) OPLS-DA results of feces samples in positive ion mode (**D**: M group vs. Con group, **H**: M group vs. M + HAPE group); (**I**) S-plot score of feces samples in negative ion mode (M group vs. Con group); (**J**) The S-plot score of feces samples in positive ion mode (M group vs. Con group); (**K**) The S-plot score of serum samples in negative ion mode (M group vs. Con group); (**L**) The S-plot score of serum samples in positive ion mode (M group vs. Con group); (**M**) The relative intensity results of metabolites in feces; (**N**) The relative intensity results of metabolites in serum. * *p* < 0.05, ** *p* < 0.01, *** *p* < 0.001, M group vs. Con group, # *p* < 0.05, ## *p* < 0.01, ### *p* < 0.01, M group vs. M + HAPE group.

To identify potential biomarkers in the Con, M, and M + HAPE groups after three weeks of treatment, the OPLS-DA score was applied. According to Figure 5A,B,E,F, the OPLS-DA of the serum revealed the complete separation of the metabolic profiles between the different groups in positive and negative ion modes. This phenomenon was also reflected in the results of the OPLS-DA of the feces (Figure 5C,D,G,H). Likewise, sevenfold cross-validation and testing with 200 rounds of permutation showed that the OPLS-DA models were robust. More importantly, the S-plot scores (Figure 5I–L) could be used to screen and identify differential metabolites. According to the standards of VIP > 1.5 and *p* < 0.05, 36 metabolites in the serum (Table S4) and 38 metabolites in the feces (Table S5) were determined. Among them, the levels of 19 metabolites were significantly altered in the serum of HL rats. These metabolites included amino acids (e.g., gamma-glutamyl-beta-cyanoalanine, arginine, valine, and tryptophan), unsaturated fatty acids (e.g., (E)-4-trimethylammoniot-2-enoate and trans-cinnamon), indol, bile acids, and other endogenous compounds. On the other hand, there were significant changes in the levels of 15 metabolites in the feces of HL rats, including amino acids, purines, unsaturated fatty acids, and other endogenous compounds. All of these metabolites were significantly reversed or regulated after the APE treatment (Figure 5M,N). The results indicated that the metabolic functions in HL rats were severely disrupted after a high-fat diet, but this could be normalized by the APE intervention. Amino acid metabolism plays an important role in this process, which might mediate changes in intercellular signaling molecules involved in processes such as lipid metabolism. These metabolic differences were considered to be potential biomarkers in the serum and feces of HL rats, which might help to elucidate the pathogenesis of HL induced by a high-fat diet and the key targets of the protective mechanism of APE against HL.

### 2.8. Untargeted Metabolomic Pathway Analysis

**Figure 6:**
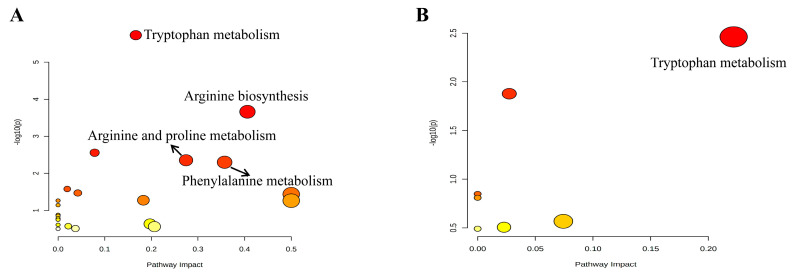
Results of pathway enrichment analysis. (**A**) Pathway enrichment analysis of metabolites in serum; (**B**) pathway enrichment analysis of metabolites in feces.

The potential biomarkers of APE for the treatment of HL may play an important role in specific metabolic pathways. Firstly, 19 and 15 metabolites from the serum and feces, respectively, were imported into the Metabo-Analyst database (https://www.metaboanalyst.ca/Metabo-Analyst/home.xhtmL, accessed on 12 December 2022) to obtain their metabolic pathways. The results of the pathway analysis revealed that the differentially expressed metabolites in the serum of the M + HAPE vs. M groups were mainly enriched in the tryptophan metabolism, arginine biosynthesis, arginine and proline metabolism, and phenylalanine metabolism pathways (*p* < 0.05). On the other hand, the main pathway involved in the differentially expressed metabolites in the feces was tryptophan metabolism (*p* < 0.05). The color and size of each circle in Figure 6A,B are based on the *p*-values and pathway effect values, respectively.

### 2.9. Quantitative Analysis of Amino Acid Metabolites in the Serum

The linear regression equations for each standard solution of amino acids were obtained, as shown in Table S6. The limits of quantification for all amino acids were between 0.5 ng/mL and 20 ng/mL, and the correlation coefficients (r) of the regressions were all greater than 0.99. Therefore, the results indicated that there was a good quantitative relationship between the concentrations of amino acid and the responses of MS. In addition, each batch of samples was followed by a QC assay at an interval of five needles, and the stability of the method was evaluated by calculating the RSD of all QCs with the peak areas of the standards. The results showed that the RSD of each amino acid was less than 5% (Table S7), indicating that this method was stable and reliable and could be used for amino-acid-targeted metabolomics analysis.

**Figure 7:**
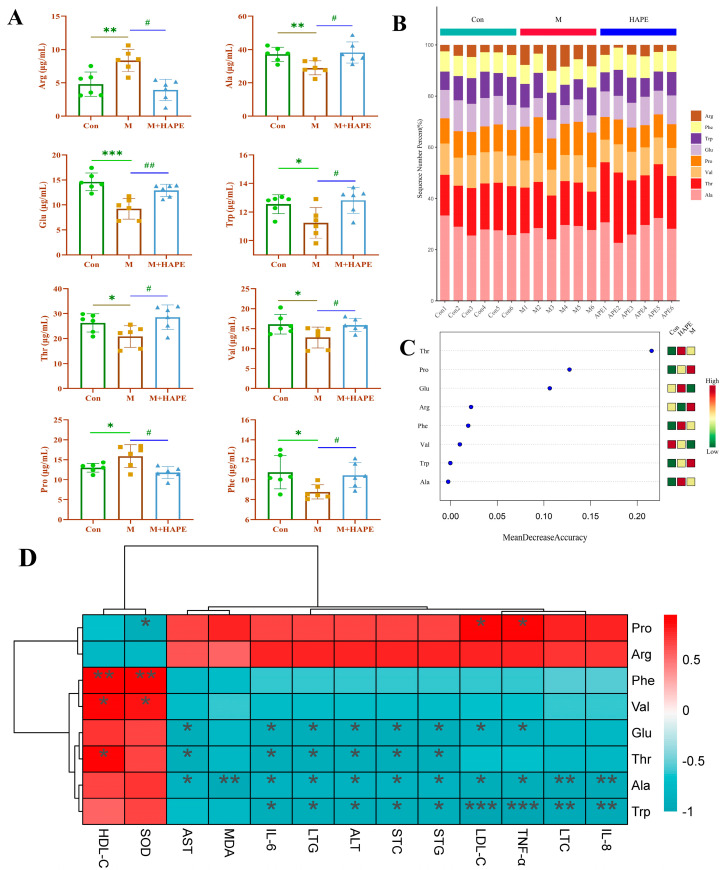
Results of APE analysis of serum-targeted amino acid metabolism in HL rats. (**A**) Effect of APE on serum levels of eight amino acids in HL rats; (**B**) Histogram of amino acid percentage stacking for different groups (n = 6); (**C**) Random forest plots for different groups of amino acids (n = 6); (**D**) Heatmap analysis of the Spearman’s correlation of eight amino acid and metabolic syndrome-related indices (positive correlation is in red and negative correlation is in blue, * *p* < 0.05, ** *p* < 0.01, *** *p* < 0.001). * *p* < 0.05, ** *p* < 0.01, *** *p* < 0.001, M group vs. Con group; # *p* < 0.05, ## *p* < 0.01, M group vs. M + HAPE group. STC: TC in serum, STG: TG in serum, LTG: TG in liver, LTC: TC in liver.

The quantitative results of LC-MS/MS showed that six amino acids were significantly lower (*p* < 0.01) and two amino acids were significantly higher (*p* < 0.01) in the M group compared with the Con group. Interestingly, the levels of alanine (Ala), glutamate (Glu), tryptophan (Trp), threonine (Thr), valine (Val), and phenylalanine (Phe) were significantly higher in the M + HAPE group, and those of arginine (Arg) and proline (Pro) were significantly lower in the M + HAPE group compared with the M group. More importantly, tryptophan, a key biomarker in the untargeted enrichment pathway, was increased one-fold in the M + HAPE group compared with the M group, and its levels were closer to those of the Con group (Figure 7A). Tryptophan is an essential amino acid that cannot be synthesized by human cells. Studies have shown that free tryptophan levels in the serum decrease with increasing degrees of cardiovascular disease. Tryptophan can influence lipid metabolism, and low levels of tryptophan have been associated with poor cardiovascular prognosis and inflammation-related pathologies. Figure 7B shows the percentage of the eight amino acids in the different groups. Figure 7C revealed the accuracy of the eight amino acids for predicting the grouping of samples. According to a combination of the experimental results and the previous literature, the levels of the eight amino acids are closely related to the lipid metabolism, glucose metabolism, and energy metabolism of the body. An abnormal lipid metabolism is the primary cause of HL. APE’s regulation of the amino acid metabolism could restore the homeostasis of the organism and improve HL. The ion pairs used for the quantitative analysis are shown in Table S8.

Spearman’s correlation analysis showed that except for SOD and HDL-C, the indicator factors were negatively correlated with Phe, Val, Glu, Thr, Ala, and Trp, but were positively correlated with Pro and Arg. Among them, Trp displayed a high correlation with the inflammatory factors IL-8, TNF-α, and IL-6 (*p* < 0.05). Ala, Thr, and Glu displayed a significantly negative correlation with TC and TG (*p* < 0.05), the key factors of lipids. Phe and Val revealed a negative correlation without significance. However, Phe and Val indicated significantly positive correlations with HDL-C and SOD (*p* < 0.05). Notably, only Ala had a significant negative correlation with MDA (*p* < 0.01), suggesting that Ala may be a key metabolite in the regulation of MDA. Arg indicated no significant correlation with any of the index factors, but there were moderating trends. Pro demonstrated a significant positive correlation with TNF-α and LDL-C (*p* < 0.05), and a significant negative correlation with SOD (*p* < 0.05). The abovementioned results indicated a correlation between the levels of the eight amino acids and metabolic disorders, including dyslipidemia, liver injury, inflammation, and oxidative stress (Figure 7D).

### 2.10. APE Regulates the mRNA Expression of Lipid-Related Factors

**Figure 8:**
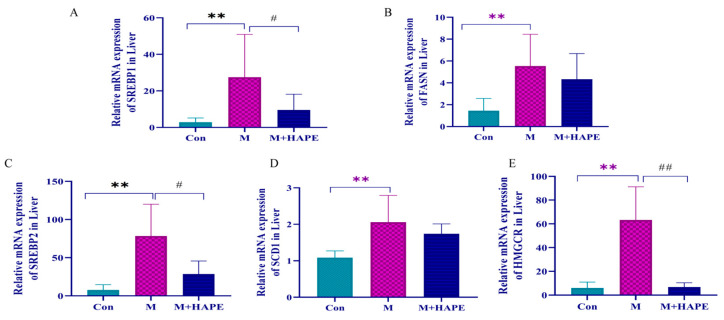
The mRNA expression results of related genes. (**A**) The mRNA expression of SREBP1; (**B**) The mRNA expression of FASN; (**C**) The mRNA expression of REBP2; (**D**) The mRNA expression of SCD1; (**E**) The mRNA expression of HMGCR. ** *p* < 0.01, M group vs. Con group, # *p* < 0.05, ## *p* < 0.01, M group vs. M + HAPE groups.

To further investigate the mechanism of APE against HL, we determined the mRNA expression levels of SREBP1, SREBP2, HMGCR, FASN, and SCD1, which are transcriptionally regulated by the SREBPS pathway. They play a key role in the synthesis of cholesterol and fatty acids in vivo. The results showed that HFD significantly upregulated the mRNA expression of five genes in the M group compared with the Con group (*p* < 0.01), and the APE treatment was able to downregulate the mRNA expression of these genes. However, the downward trend was not completely consistent. APE significantly downregulated the mRNA expression of SREBP1, SREBP2, and HMGCR (*p* < 0.05), while the mRNA levels of FASN and SCD1 were not significantly downregulated (*p* > 0.05). A series of results suggested that the mechanism of APE for HL may act by interfering with the SREBP/HMGCR signaling pathway. The results are shown in Figure 8.

## 3. Discussion

HL is considered to be one of the highest risk factors for cardiovascular disease [14]. The first-line drugs commonly used today include three classes: statins, fibrates and niacin. Patients with HL who take these synthetic drugs for a long time may become dependent on these drugs, and they have toxic side effects on the body [15]. Therefore, developing natural active drugs to replace chemically synthesized drugs for HL can help reduce the damage to the body. Bioactive peptides, due to their safety and efficiency, are widely used for the treatment of diseases. In this study, APE was prepared through an enzymatic process, and its structure was characterized using various analytical methods. In addition, a rat model of HFD-induced HL was established to evaluate the anti-HL activity of APE. The results showed that APE not only significantly reduced serum TG, TC, and LDL-C levels (*p* < 0.05), but also increased serum HDL-C levels (*p* < 0.05). Moreover, APE reduced the accumulation of lipids in the liver; decreased the levels of AST, ALT, and MDA; and increased the level of SOD, thereby reducing the liver injury caused by oxidative stress. The results of an ELISA showed that APE improved the inflammatory response in HL rats by reducing serum IL-6, IL-8, and TNF-α levels.

Diseases inevitably lead to metabolic disturbances in vivo, and regulation of the levels of metabolites, as direct players in the metabolic pathways, can also reflect the risk of disease [16]. To further explore the beneficial role and potential mechanisms of APE in the treatment of HL, a metabolic profiling strategy combining untargeted and targeted metabolomic techniques was used. It was suggested that HFD could indeed disrupt the metabolic profile in the serum and feces, and that APE could reverse this. The relative intensities of 19 metabolites in the serum and 15 metabolites in the feces were significantly changed (*p* < 0.05). The results of the pathway enrichment analysis suggested that the mechanism of action of APE against HL may mainly be related to the tryptophan metabolism, arginine biosynthesis, arginine and proline metabolism, and phenylalanine metabolism pathways. An increasing number of studies have shown that amino acid metabolism plays an important role in HL.

In line with this, we performed a targeted quantitative analysis of eight amino acids. The results showed that the levels of Trp, Ala, Glu, Thr, Val, and Phe increased, and levels of Pro and Arg decreased after the APE treatment. A correlation analysis of the eight amino acids with the indicators revealed that except for SOD and HDL-C, the indicators were negatively correlated with Phe, Val, Glu, Thr, Ala, and Trp, but positively correlated with Pro and Arg. Among them, Trp was highly correlated with the inflammatory factors IL-8, TNF-α, and IL-6. Studies have shown that Trp prevents lipopolysaccharides from entering the bloodstream by repairing the damage to the intestinal barrier induced by high-density lipoprotein cholesterol (HFD), which in turn alleviates hepatic steatoinflammation by interfering with the TLR/NF-κB signaling pathway and inhibiting the release of TNF-α, IL-6, and interleukin-1β (IL-1β) [17,18]. HE staining proved that APE significantly ameliorated HFD-induced hepatic inflammation and degeneration in rats, which may be related to the indirect intervention of tryptophan. In addition, Ala, Thr, and Glu were significantly negatively correlated with TC and TG, the key factors of blood lipids. The accumulation of hepatic fat has been reported to promote the release of the pro-inflammatory factors TNF-α, IL-6, and IL-8, leading to hepatic inflammation and cellular damage [19]. It was found that the levels of TC, TG, TNF-α, IL-6, and IL-8 were significantly elevated in the M group compared with the Con group, and were significantly decreased by the APE treatment. At the same time, APE modulated the levels of Ala, Thr, and Glu in HL rats. Moreover, Phe and Val indicated significant positive correlations with HDL-C and SOD. Notably, SOD is an antioxidant enzyme that scavenges reactive oxygen species in vivo. Together with MDA, a product of lipid peroxidation, the antioxidant capacity of the body can be determined by the levels of SOD [20]. After the APE intervention, the levels of SOD increased, and those of MDA decreased in HL rats. Therefore, we hypothesized that APE enhances antioxidant enzymes’ activity by modulating metabolically disturbed Phe and Val.

Currently, research has gained more insight into the mechanisms of amino acid-derived metabolite-induced HL diseases. Yan et al. proved that amino acid inhibited the activation of mTOR and proinflammatory cytokines and reduced the expression of cholesterol metabolism proteins [21]. Proline plays a key role in lipid metabolism and oxidative stress in hepatocytes. Threonine could effectively alleviate the inflammatory response in HL rats by decreasing the levels of inflammatory factors [22]. Valine, an antioxidant, could improve endothelial dysfunction by decreasing the levels of reactive oxygen species, thereby reducing oxidative stress in HL rats [23]. Elevated levels of alanine and glutamate may improve lipid metabolism, reduce oxidative stress and inflammation, and facilitate the treatment of HL [24,25].

To further investigate the mechanism of APE against HL, the expression levels of genes related to lipid metabolism in HL rats were measured. The results showed that APE inhibited the expression of SREBP1, SREBP2, HMGCR, FASN, and SCD1, and promoted cholesterol conversion and fatty acid synthesis. Taken together, these results suggested that APE improves HL by modulating amino acid metabolism in the metabolites of the serum and feces, mediating the SREBP/HMGCR signaling pathway and reducing the levels of oxidative stress and inflammation.

This study elucidated the mechanisms of APE in the treatment of HL via multiple pathways. The findings may provide an experimental basis for the development of APE as a potential functional food or food additive against HL.

## 4. Materials and Methods

### 4.1. Reagents and Materials

*E. sinensis* was purchased from Hebei Hongsen Pharmaceutical Co., Ltd. (Shijiazhuang, China). Pepsin (2500 U/mg) and trypsin (5000 U/mg) were all obtained from Shanghai Sinopharm Reagent Group Co., Ltd. (Shanghai, China). Simvastatin was supplied by Merck Pharmaceuticals Ltd. (Hangzhou, China). The kits of total cholesterol (TC), triglyceride (TG), high-density lipoprotein cholesterol (HDL-C), and low-density lipoprotein cholesterol (LDL-c), alanine amino transferase (ALT), and aspartate amino transferase (AST), superoxide dismutase (SOD), and methane dicarboxylic aldehyde (MDA) were purchased from Nanjing Jiancheng Bioengineering Institute (Nanjing, China). Enzyme-linked immunosorbent assay (ELISA) kits of tumor necrosis factor alpha (TNF-α), interleukin-8 (IL-8), and interleukin-6 (IL-6) were obtained from Shanghai Zhe Ke Biological Technology Co., Ltd. (Shanghai, China). HPLC-grade methanol, acetonitrile, and formic acid (FA) were purchased from Thermo Fisher Scientific (Fair Lawn, NJ, USA), and pure water for analysis was purchased from Watson Group Co., Ltd. (Jinan, China). The high-fat diet (HFD), including basic feed 65%, lard 15%, cholesterol 5%, egg yolk 10%, and sodium bile 5%, was provided by Beijing Huafukang Biotechnology Co., Ltd. (Beijing China). 

L-Valine was obtained from Sigma-Aldrich (Shanghai, China). L-Glutamine was obtained from Aladdin (Shanghai, China). L-Alanine, L-Proline, L-Threonine, L-Phenylalanine, L-Arginine, and L-Tryptophan were all obtained from Sinopharm Chemical Reagent Co., Ltd. (Shanghai, China). DL-Tryptophan-2,3,3,-d3 was obtained from C/D/N Isotopes Inc. (Pointe-Claire, QC, Canada).

### 4.2. Preparation of APE

*E. sinensis* was dissolved in deionized water to a concentration of 10% (*w*/*v*). Firstly, the sample was denatured at 90 °C for 10 min and cooled to 40 °C before the pH was adjusted to 2.0 with 1.0 M HCl. The solution was then added to pepsin (1.0%) and stirred for 1.0 h. The pH was then adjusted to 8.0 with 1.0 M NaOH, and trypsin (1.0% of drug) was added and stirred for 3.0 h. Finally, the enzyme solution was heated in boiling water for 15 min to inactivate the enzyme. The mixture was then centrifuged at 5000 rpm for 10 min. Most importantly, the resulting supernatant was separated into different components using an ultrafiltration membrane filter processing system (GF3000, Shanghai Moso Scientific Equipment Co., Ltd., Shanghai, China). Fractions with molecular weights between 150 and 3000 Da were lyophilized to obtain APE, and stored at −20 °C until use [14].

### 4.3. Physicochemical Properties of APE

The peptide content of APE was measured by the Folin phenol reagent method [26]. The sugar content of APE was detected at 490 nm using the phenol–sulfuric acid method [27]. The pH of APE was measured using a pH meter (S400-B, METTLER TOLEDO, Columbus, OH, USA). In addition, the maximum absorption wavelength of APE was measured using a UV spectrophotometer. Finally, the peptide sequences of APE were analyzed for identification using Easy nLC1200/Q Exactive Plus. Separation was performed on a Waters ACQUITY UPLC Peptide BEH C18 column (2.1 × 100 mm, 1.8 μm). The mobile phase was made up of 0.1% formic acid (A) and acetonitrile (B). The elution gradient was set as follows: 0–3 min with 2–8% B; 3–42 min with 8–20% B; 42–48 min with 20–35% B; 48–49 min with 35–100% B; 49–60 min at 100 B%. The mass spectrometry conditions comprised an ESI+ detection mode with data-dependent scanning and full-scan acquisition (*m*/*z* 200–1600) in an orbital trap with a resolution of 70,000 (AGC 3e6). The parent ions of the first 20 isolated peptide signals (charge states ≥ +1) were fragmented via high-energy collision (HCD) with a normalized collision energy (NCE) of 28.0. The capillary’s temperature was 275 °C and the spray’s voltage was 1800 V. The maximum filling times were set to 50 ms and 45 ms for the full and MS-MS scans, respectively, and the dynamic exclusion time was set to 30 s.

### 4.4. Antihyperlipidemic Effect of APE

#### 4.4.1. Establishment of the HL Rat Model and Treatments

Fifty male Sprague Dawley rats with a weight of 180–200 g were purchased from Jinan Pengyue Experimental Animal Co., Ltd. (Jinan, China, SYXK(LU)2018-0003). All animals were housed for 1 week under standard conditions (temperature, 24 ± 2 °C; humidity, 55–60%; 12/12 h light/dark cycle). Subsequently, all rats were randomly divided into the control group (Con, 10 rats) and the HL group (40 rats) according to their body weight. The Con group was fed with a normal diet and the HL group was fed with a high-fat diet (HFD). After 8 weeks, all rats fed the HFD were divided into four groups, namely the model group (M, n = 10), the simvastatin group (Sim, 5 mg/kg/d, n = 10), the high-dose APE group (M + HAPE, 100 mg/kg/d, n = 10), and the low-dose APE group (M + LAPE, 25 mg/kg/d, n = 10). The drugs were delivered by intragastric (i.g.) administration. Except for the Con group, the rats were still fed the HFD for 3 weeks of treatment [14].

#### 4.4.2. Collections and Preparation of Biological Samples

The rats were placed in metabolic cages, and fresh feces were collected for 24 h. At the end of the experiment, the rats were euthanized with 10% pentobarbital sodium delivered intraperitoneally. Blood was taken from the abdominal aorta, and the serum was prepared. Samples of the liver tissues were fixed with 4% paraformaldehyde immediately after collection of the blood. Plasma, serum, and the remaining liver tissues were stored at −80 °C prior to analysis.

#### 4.4.3. Serum Biochemical Indicator Test and Histopathological Analysis

Serum TG, TC, HDL-C, LDL-C, ALT, AST, SOD, and MDA levels were analyzed using commercial kits. Then, hematoxylin and eosin (H&E) were used for histopathological analysis of liver sections from 4% paraformaldehyde-fixed samples. Additionally, lipid accumulation in the liver was detected via Oil Red O staining.

#### 4.4.4. ELISA

Serum IL-6, IL-8, and TNF-α levels were measured in each group using an ELISA, which was performed according to the protocol described in the kit.

### 4.5. Untargeted Metabolomics Analysis of Serum and Faeces

#### 4.5.1. Preparation of the Samples

Serum and feces samples were prepared using the protein precipitation method. For the serum samples, 800 μL of methanol was added to 400 μL of the serum, and the mixture was vortexed for 1 min and centrifuged at 10,000 rpm for 10 min. The supernatant was then collected and dried with N2 at 4 °C. The residue was dissolved with 200 μL of methanol and centrifuged at 14,000 rpm for 10 min. Finally, the supernatant was collected for analysis. Feces stored at −80 °C were freeze-dried and crushed, then 100 mg of the powders was mixed with 1 mL of 50% cold ethanol, and the proteins were extracted via ultrasonication for 30 min and centrifuged at 10,000 rpm for 10 min. The supernatant was collected and evaporated with N_2_ at 4 °C. The residue was dissolved with 200 µL of methanol and centrifuged at 14,000 rpm to obtain the supernatant of the feces for further analysis. To obtain quality control (QC) samples, 10 µL of the solution of each serum and each feces sample was mixed and labeled as the serum QC samples and feces QC samples, respectively.

#### 4.5.2. UPLC-Q-Exactive HRMS/MS Analysis Conditions

LC analysis was performed on a DIONEX Ultimate 3000 UHPLC system (Thermo Fisher Scientific, Waltham, MA, USA). Separation was performed on an ACQUITY UPLC BEH HILIC column (1.7 μm, 2.1 × 150 mm) with a column temperature of 35 °C. The flow rate was 0.3 mL/min, and the injection volume was 3 μL. The mobile phase was composed of water containing 0.1% formic acid (A) and acetonitrile (B). The elution parameters were 0–5.0 min, 5% A; 5.0–6.0 min, 5–20% A; 6.0–10 min, 20–25% A; 10–13 min, 25–40% A; 13–16 min, 40% A; 16–16.5 min, 40–45% A; 16.5–20 min, 5% A.

HRMS/MS spectra were obtained using Q-Exactive Focus Orbitrap MS (Thermo Fisher, Waltham, MA, USA). All samples were analyzed in negative and in positive ion modes. The ion source parameters were set as follows: Spray voltage, 3.8/3.5 kV (+/−); Sheath gas flow rate, 30 arb; Aux gas flow rate, 10 arb; Capillary temperature, 320 °C; Scan modes, full MS resolution, 70,000; dd-MS^2^ resolution, 17,500; Scan range, *m*/*z* 70–1050; Collision energy, 30%.

#### 4.5.3. Data Processing and Analysis

The raw MS data were processed with Compound Discoverer 3.1 software (Thermo Fisher Scientific, MA, USA) for peak alignment, normalization, and correction to obtain reliable data matrixes, including *m*/*z*, relative peak intensity, and retention time. The resultant data matrices were imported into SMICA_14.0 software (Umetrics, Umea, Sweden) for principal component analysis (PCA) and orthogonal partial least-squares discriminant analysis (OPLS-DA). Differential metabolites were diagnosed using multiple indicators, consisting of variable importance in projection (VIP) ≥1.5 (generated in the OPLS-DA mode) and *p* < 0.05 (formed from relative intensity). Then, differential metabolites were imported into the Human Metabolome Database (https://hmdb.ca/, accessed on 15 February 2023) to obtain precise information (compound name, molecular weight, and code). In addition, the metabolic pathways of the differential metabolites were analyzed using the Kyoto Encyclopedia of Genes and Genomes (KEGG) Pathway database (http://www.kegg.jp/kegg/pathway.html, accessed on 17 February 2023). Finally, the interrelationship between feces metabolites, serum metabolites, and biochemical indicators was revealed using the Spearman’s correlation analysis method (https://bioincloud.tech/, accessed on 18 February 2023).

### 4.6. Targeted Metabolomics Analysis of Serum Amino Acids

#### 4.6.1. Samples Preparation

Serum samples were extracted in 400 μL of 10% formic acid in methanol–water (1:1, *v*/*v*), and vortexed for 30 s, before being centrifuged at 12,000 rpm and 4 °C for 5 min. An appropriate amount of supernatant was taken and 10% formic acid was added in methanol–water (1:1, *v*/*v*) to dilute 10 times, before the mixture was vortexed for 30 s. Some 100 μL of supernatant was taken, and 100 μL Trp-d3(10 ng/mL) was added and vortexed for 30 s. The supernatant was passed through a 0.22 μm filter and added to the LC-MS bottle. The QC samples were mixed 1:1 by volume from the serum samples to be tested, to determine whether the quality of the assay data was acceptable.

Calculation formula:

$$\begin{array}{l} \mathrm{The}\mathrm{content}\mathrm{of}\mathrm{sample}=C\times (0.4+\mathrm{Amount}/1000)\times 2\times 10/\mathrm{Amount}\\ \mathrm{The}\mathrm{unit}\mathrm{of}\mathrm{content}:\mu g/\mathrm{mL}\\ \mathrm{The}\mathrm{unit}\mathrm{of}C:\mathrm{ng}/\mathrm{mL}\\ \mathrm{The}\mathrm{unit}\mathrm{of}\mathrm{amount}:\mu L \end{array}$$

#### 4.6.2. MRM-MS Analysis Conditions

The LC analysis was performed using Jasper HPLC Liquid chromatography (AB SCIEX, USA). A ZORBAX Eclipse XDB-C18 column (4.6 × 150 mm, Agilent, Santa Clara, CA, USA) was used with an injection volume of 5 μL. The mobile phases were A-10% methanolic water (containing 0.1% formic acid) and B-50% methanolic water (containing 0.1% formic acid). The gradient elution conditions were 0–6.5 min, 10–30% B; 6.5–7 min, 30–100% B; 7–18 min, 100% B; 18–18.5 min, 100–110% B; 18.5–21 min, 10% B; 0–8 min, flow rate 0.3 mL/min; 8.5–21 min, and flow rate 0.4 mL/min.

Mass spectrometric detection of metabolites was performed using AB4500MD (AB SCIEX, Redwood City, CA, USA). The electrospray ionization (ESI) source conditions in positive ion mode were as follows: ion source temperature, 500 °C; ion source voltage, 5500 V; collision gas, 6 psi; air curtain gas, 30 psi; and nebulizer gas and auxiliary gas, both 50 psi. MRM mode was then used to detect the ion pairs.

#### 4.6.3. Data Processing and Analysis

The peak areas and retention times of the amino acids were extracted using Multiquant software (https://sciex.com/products/software/multiquant-software, accessed on 28 February 2023). The retention times were corrected according to the standards of amino acids, and then the metabolites were identified.

### 4.7. The mRNA Expression Analysis of Lipid-Related Factors

To briefly illustrate the effect of APE on lipid accumulation, the mRNA expressions of five key factors were determined via RT-PCR. Firstly, Total RNA in liver tissues from Con, M and M + HAPE groups was extracted using TRIzol RT-PCR preparing reagents (Invitrogen, Carlsbad, CA, USA), according to the instructions of the manufacturer. A transcriptor One-Step RT-PCR Kit was used for qPCR, along with specific primers for glyceraldehyde phosphate dehydrogenase (GAPDH, FWD: 5′-CTGGAGAAACCTGCCAAGTATG-3′, REV: 5′-GGTGGAAGAATGGGAGTTGCT-3′), hydroxymethylglutaryl-CoA reductase (HMGCR, FWD: 5′-GCAGGACGCAACCTCTACATC-3′, REV: 5′-CACCACCTTGGCTGGAATGA-3′), sterol regulatory element-binding proteins (SREBP1, FWD: 5′-TTGAGGATAACCAGGTGAAAGCC-3′, REV: 5′-CGAAGCATCAGAGGGAGTGA-3′), sterol regulatory element-binding proteins (SREBP2, FWD: 5′-ATCCTCGCAGGTACAGCCAGTT-3′, REV: 5′-GGGTTGGTACTTGAAGGGCG-3′), SCD1(FWD: 5′-CTGGAGATGGGAGCCACAAGA-3′, REV: 5′-ACATTCCGATAGCATTATCCAGTAG-3′), fatty acid synthase (FASN, FWD: 5′-CTGGAGCGTGAGCACAACCTG-3′, REV: 5′-GTGTGGAGTCCGTCAGCTCAT-3′), and Stearoyl-CoA desaturase1 (SCD1, FWD: ACCTGGCTGGTGAATAGTGC, REV: TGACATATGGAGAGGGTCA). The quantitative RT-PCR testing was performed using an ABI ViiA7 Real-Time PCR System with cycling conditions of 50 °C for 10 min and 95 °C for 1.0 min, which was then repeated 40 times at 95 °C for 15 s each time; for re-processing, the conditions were 60 °C for 30 s. The data were normalized to the mRNA levels of GAPDH and quantified using the 2^−ΔΔCt^ method.

### 4.8. Statistical Analysis

The statistical analysis was performed using SPSS 22.0 software. All data were expressed as Mean ± standard deviation (SD). Student’s *t*-test was used to assess statistical significance between the two groups. *p* < 0.05 and *p* < 0.01 were chosen to define statistical significance. The statistical analyses were performed and figures were obtained using GraphPad Prism 8.0 software.

## 5. Conclusions

In conclusion, APE reduced blood lipids and improved oxidative stress and inflammatory responses in HL rats. The results of an untargeted metabolomic analysis indicated that APE had a modulating effect on HL-induced disturbed metabolism in the serum and feces. The results of the pathway analysis showed that the effects of APE on HL were related to tryptophan metabolism, phenylalanine metabolism, arginine biosynthesis, and purine metabolism. In particular, amino acid metabolism was altered, and the effect of APE on HL was accompanied by increased levels of serum tryptophan, alanine, glutamate, threonine, valine, and phenylalanine, and decreased levels of arginine and proline, as determined by the targeted amino acid metabolomic analysis. In addition, APE promoted the conversion of cholesterol and fatty acid synthesis by inhibiting the expression of hepatic SREBP1, SREBP2, HMGCR, FASN, and SCD1. These results provide new evidence for the possible molecular mechanisms and targets of APE against HL for clinical diagnosis and treatment. However, the present study still has some limitations. Although we summarized the changes in the levels of eight amino acids in HL rats, more in vivo functional validation is still needed. In short, our experimental results confirmed the application value of the bioactive peptide APE and provide theoretical support for the design and production of lipid-lowering functional foods.

## Data Availability

Most of the data used during the preparation of the manuscript are included in the Results and Discussion sections. However, for any additional details of the procedures and the original raw files, please contact the corresponding authors.

## Supplementary Materials

The following supporting information can be downloaded at: https://www.mdpi.com/article/10.3390/molecules28207049/s1, Table S1: Results of peptides and sugars in APE; Table S2: The results of pH in APE; Table S3: Peptide sequence composition of APE (Top 60); Table S4: Identified potential biomarkers in serum regulated by APE; Table S5: Identified potential biomarkers in feces regulated by APE; Table S6: Linear regression equation and quantitative limits of amino acids; Table S7: Stability of various targeting substances in QC samples; Table S8: Quantitative ion pairs of amino acids. Figure S1: The base peak plots of QC samples in the positive and negative ions for serum and feces samples. Figure S2: Results of PLS-DA analysis based on SCIMA_P 14.0.

## Sample Availability

Samples of the compounds are available from the authors.
