# Supplementary material for: Untargeted and Targeted Metabolomics Reveal the Active Peptide of Eupolyphaga sinensis Walker against Hyperlipidemia by Modulating Imbalance in Amino Acid Metabolism
Source: Molecules. 2023 Oct 12;28(20):7049. doi: 10.3390/molecules28207049 (PMC10609387; doi:10.3390/molecules28207049)
Supplement: Supplementary file 1 [file molecules-28-07049-s001.zip › molecules-2621655-supplementary.pdf]

**Table S1.** The results of peptide and Sugar in APE

| Projects | Regression curve | Decisive factor (R2) | Contents (mg/g) | Average content (mg/g) | SD |
|---|---|---|---|---|---|
| Peptide | Y=2.1283X+0.0074 | 0.9991 | 633.14<br>631.05<br>640.57<br>599.38<br>632.59<br>604.63 | 623.56 | 17.10 |
| Sugar | Y=7.7X-0.1006 | 0.9936 | 34.67<br>36.03<br>35.66<br>34.12<br>35.69<br>35.17 | 35.22 | 0.72 |

**Table S2.** The results of pH in APE

| Number of measurements | pH | Average pH | SD |
|---|---|---|---|
| 1 | 7.96 | 8.12 | 0.26 |
| 2 | 8.32 | | |
| 3 | 8.44 | | |
| 4 | 7.76 | | |
| 5 | 7.99 | | |
| 6 | 8.25 | | |

**Table S3**. Peptide sequence composition of APE (Top 60)

| Peptide | -10lgP | Mass | Length | ppm | m/z | RT | Area Sample 2 | Accession |
|---|---|---|---|---|---|---|---|---|
| AVFPSIVGR | 25.71 | 944.5443 | 9 | 1.1 | 473.28 | 37.51 | 1.40E+10 | tr\|Q3HNA6\|Q3HNA6_RETFL:tr\|A0A067QL86\|A0A067QL86_ZOONE:tr\|A0A2J7RHY5\|A0A2J7RHY5_9NEOP:tr\|A0A2Z5RE76\|A0A2Z5RE76_9NEOP:tr\|A0A067RMN2\|A0A067RMN2_ZOONE:tr\|A0A2P8Z2J3\|A0A2P8Z2J3_BLAGE:tr\|A0A2R4CIH4\|A0A2R4CIH4_PERAM:tr\|A0A2J7PLV1\|A0A2J7PLV1_9NEOP:tr\|A0A067R416\|A0A067R416_ZOONE:tr\|A0A2J7QYF0\|A0A2J7QYF0_9NEOP |
| AVFPSIVGRP | 29.9 | 1041.597 | 10 | 1.1 | 1042.6055 | 41.7 | 8.72E+09 | tr\|Q3HNA6\|Q3HNA6_RETFL:tr\|A0A067QL86\|A0A067QL86_ZOONE:tr\|A0A2J7RHY5\|A0A2J7RHY5_9NEOP:tr\|A0A2Z5RE76\|A0A2Z5RE76_9NEOP:tr\|A0A067RMN2\|A0A067RMN2_ZOONE:tr\|A0A2P8Z2J3\|A0A2P8Z2J3_BLAGE:tr\|A0A2R4CIH4\|A0A2R4CIH4_PERAM:tr\|A0A2J7PLV1\|A0A2J7PLV1_9NEOP:tr\|A0A067R416\|A0A067R416_ZOONE:tr\|A0A2J7QYF0\|A0A2J7QYF0_9NEOP |
| EITALAPSTIK | 32.07 | 1142.6547 | 11 | 0 | 1143.662 | 35.78 | 8.13E+09 | tr\|Q3HNA6\|Q3HNA6_RETFL:tr\|A0A067QL86\|A0A067QL86_ZOONE:tr\|A0A2J7RHY5\|A0A2J7RHY5_9NEOP:tr\|A0A2Z5RE76\|A0A2Z5RE76_9NEOP |
| TAPGIVASANTAGGF | 40.38 | 1332.6674 | 15 | 2.1 | 1333.6775 | 47.56 | 6.62E+09 | tr\|Q5H799\|Q5H799_9NEOP |
| VAAPVAVAAPVA | 20.12 | 1034.6124 | 12 | 1.7 | 518.3144 | 48.12 | 5.87E+09 | tr\|A0A2P8YBM8\|A0A2P8YBM8_BLAGE:tr\|A0A2P8YBL7\|A0A2P8YBL7_BLAGE:tr\|A0A2P8YBP8\|A0A2P8YBP8_BLAGE |
| GIITNWDDMEK | 37.67 | 1320.6021 | 11 | 1.7 | 1321.6116 | 43.64 | 5.19E+09 | tr\|Q3HNA6\|Q3HNA6_RETFL:tr\|A0A067QL86\|A0A067QL86_ZOONE:tr\|A0A2J7RHY5\|A0A2J7RHY5_9NEOP:tr\|A0A2Z5RE76\|A0A2Z5RE76_9NEOP |
| NISGWLEK | 26.22 | 945.4919 | 8 | -0.2 | 946.499 | 27.46 | 4.69E+09 | tr\|A0A2P9A976\|A0A2P9A976_BLAGE:tr\|A0A2P8XQ42\|A0A2P8XQ42_BLAGE |
| HQGVMVGMGQK | 42.34 | 1170.5638 | 11 | 1.2 | 1171.5725 | 9.31 | 3.89E+09 | tr\|Q3HNA6\|Q3HNA6_RETFL:tr\|A0A067QL86\|A0A067QL86_ZOONE:tr\|A0A2J7RHY5\|A0A2J7RHY5_9NEOP:tr\|A0A2Z5RE76\|A0A2Z5RE76_9NEOP:tr\|A0A067RMN2\|A0A067RMN2_ZOONE:tr\|A0A2P8Z2J3\|A0A2P8Z2J3_BLAGE:tr\|A0A2R4CIH4\|A0A2R4CIH4_PERAM:tr\|A0A2J7PLV1\|A0A2J7PLV1_9NEOP |
| AVFPSIVG | 17.49 | 788.4432 | 8 | 0.2 | 789.4507 | 53.95 | 3.87E+09 | tr\|Q3HNA6\|Q3HNA6_RETFL:tr\|A0A067QL86\|A0A067QL86_ZOONE:tr\|A0A2J7RHY5\|A0A2J7RHY5_9NEOP:tr\|A0A2Z5RE76\|A0A2Z5RE76_9NEOP:tr\|A0A067RMN2\|A0A067RMN2_ZOONE:tr\|A0A2P8Z2 |

<table>
<tr><td></td><td></td><td></td><td></td><td></td><td></td><td></td><td></td><td>J3|A0A2P8Z2J3_BLAGE:tr|A0A2R4CIH4|A0A2R4CIH4_PERAM:tr|A0A2J7PLV1|A0A2J7PLV1_9NEOP:tr|A0A067R416|A0A067R416_ZOONE:tr|A0A2J7QYF0|A0A2J7QYF0_9NEOP</td></tr>
<tr><td>MVYPDFK</td><td>22.95</td><td>898.4258</td><td>7</td><td>0.3</td><td>899.4333</td><td>25.16</td><td>3.86E+09</td><td>tr|A0A2J7QRJ1|A0A2J7QRJ1_9NEOP:tr|A0A2P9A976|A0A2P9A976_BLAGE:tr|A0A067RA03|A0A067RA03_ZOONE</td></tr>
<tr><td>VAAAPVAKVAAVAAP</td><td>19.69</td><td>1304.7815</td><td>15</td><td>1.8</td><td>653.3992</td><td>33.56</td><td>3.45E+09</td><td>tr|A0A2P8Z7U5|A0A2P8Z7U5_BLAGE</td></tr>
<tr><td>IWHHTFY</td><td>25.06</td><td>1002.4712</td><td>7</td><td>-0.1</td><td>1003.4784</td><td>15.34</td><td>3.32E+09</td><td>tr|Q3HNA6|Q3HNA6_RETFL:tr|A0A067QL86|A0A067QL86_ZOONE:tr|A0A2J7RHY5|A0A2J7RHY5_9NEOP:tr|A0A2Z5RE76|A0A2Z5RE76_9NEOP:tr|A0A067RMN2|A0A067RMN2_ZOONE:tr|A0A2P8Z2J3|A0A2P8Z2J3_BLAGE:tr|A0A2R4CIH4|A0A2R4CIH4_PERAM:tr|A0A2J7PLV1|A0A2J7PLV1_9NEOP:tr|A0A067R416|A0A067R416_ZOONE:tr|A0A2J7QYF0|A0A2J7QYF0_9NEOP</td></tr>
<tr><td>IHFGPSGK</td><td>27.49</td><td>841.4446</td><td>8</td><td>0.3</td><td>842.4521</td><td>9.12</td><td>3.05E+09</td><td>tr|A0A2J7QRJ1|A0A2J7QRJ1_9NEOP:tr|A0A2P9A976|A0A2P9A976_BLAGE:tr|A0A2P8XQ42|A0A2P8XQ42_BLAGE</td></tr>
<tr><td>AAPALAHAPVAY</td><td>31.72</td><td>1150.6134</td><td>12</td><td>1.3</td><td>576.3147</td><td>26.77</td><td>2.87E+09</td><td>tr|A0A2J7Q400|A0A2J7Q400_9NEOP</td></tr>
<tr><td>VAAPVAY</td><td>16.4</td><td>689.3748</td><td>7</td><td>0.8</td><td>690.3826</td><td>22.25</td><td>2.68E+09</td><td>tr|A0A2P8YBK0|A0A2P8YBK0_BLAGE:tr|A0A2J7Q400|A0A2J7Q400_9NEOP:tr|A0A2J7Q3Z2|A0A2J7Q3Z2_9NEOP:tr|A0A067QWG3|A0A067QWG3_ZOONE:tr|A0A2P8Z7V6|A0A2P8Z7V6_BLAGE:tr|A0A067QUK4|A0A067QUK4_ZOONE:tr|A0A2J7QCE5|A0A2J7QCE5_9NEOP:tr|A0A2P8YNR5|A0A2P8YNR5_BLAGE:tr|A0A2J7Q412|A0A2J7Q412_9NEOP:tr|A0A2J7Q3Z3|A0A2J7Q3Z3_9NEOP:tr|A0A2J7PG72|A0A2J7PG72_9NEOP:tr|A0A2P8Y889|A0A2P8Y889_BLAGE:tr|A0A067RB44|A0A067RB44_ZOONE:tr|A0A2J7RR10|A0A2J7RR10_9NEOP</td></tr>
<tr><td>VPTSVPVLGK</td><td>33.9</td><td>995.6015</td><td>10</td><td>0.2</td><td>996.609</td><td>27.89</td><td>2.67E+09</td><td>tr|A0A2J7QTD8|A0A2J7QTD8_9NEOP:tr|A0A2P8XYU4|A0A2P8XYU4_BLAGE</td></tr>
<tr><td>VAPEEHPILL</td><td>32.86</td><td>1116.6179</td><td>10</td><td>0</td><td>1117.6252</td><td>34.69</td><td>2.45E+09</td><td>tr|Q3HNA6|Q3HNA6_RETFL:tr|A0A067QL86|A0A067QL86_ZOONE:tr|A0A2J7RHY5|A0A2J7RHY5_9NEOP:tr|A0A2Z5RE76|A0A2Z5RE76_9NEOP</td></tr>
<tr><td>LFVSLEQK</td><td>23.63</td><td>962.5436</td><td>8</td><td>0.2</td><td>963.5511</td><td>28.92</td><td>2.26E+09</td><td>tr|A0A2J7QRJ1|A0A2J7QRJ1_9NEOP:tr|A0A2P9A976|A0A2P9A976_BLAGE:tr|A0A2P8XQ42|A0A2P8XQ42_BLAGE:tr|A0A067RA03|A0A067RA03_ZOONE</td></tr>
<tr><td>LIDDHFLFK</td><td>29.86</td><td>1146.6073</td><td>9</td><td>2</td><td>574.3121</td><td>44.94</td><td>2.22E+09</td><td>tr|A1KY39|A1KY39_PERAM:tr|B9VAT1|B9VAT1_BLAGE:tr|Q2HZF2|Q2HZF2_BLAGE:tr|D3JUE7|D3</td></tr>
</table>

| | | | | | | | | |
|---|---|---|---|---|---|---|---|---|
| ISLTTQLEDTK | 36.2 | 1247.6609 | 11 | 1 | 1248.6694 | 29.66 | 2.08E+09 | JUE7_PERAM:tr|A0A067RAL9|A0A067RAL9_ZOONE:tr|A0A2J7R607|A0A2J7R607_9NEOP tr|A0A2J7QRJ1|A0A2J7QRJ1_9NEOP:tr|A0A2P9A976|A0A2P9A976_BLAGE:tr|A0A2P8XQ42|A0A2P8XQ42_BLAGE |
| VNELTTINVNLASAK | 35.59 | 1585.8676 | 15 | 1.1 | 793.9419 | 48.96 | 2.06E+09 | tr|A0A140KPR3|A0A140KPR3_BLAGE |
| TTLEASLH | 26.83 | 870.4447 | 8 | 0.2 | 871.4521 | 13.39 | 2.02E+09 | tr|A0A2P0XJ16|A0A2P0XJ16_PERAM:tr|A0A2P8ZN67|A0A2P8ZN67_BLAGE |
| ANTVLSGGTTMYPGIADR | 37.43 | 1822.8883 | 18 | 0.3 | 912.4517 | 45.41 | 1.84E+09 | tr|Q3HNA6|Q3HNA6_RETFL:tr|A0A067QL86|A0A067QL86_ZOONE:tr|A0A2J7RHY5|A0A2J7RHY5_9NEOP:tr|A0A2Z5RE76|A0A2Z5RE76_9NEOP:tr|A0A067RMN2|A0A067RMN2_ZOONE:tr|A0A2P8Z2J3|A0A2P8Z2J3_BLAGE:tr|A0A2R4CIH4|A0A2R4CIH4_PERAM:tr|A0A2J7PLV1|A0A2J7PLV1_9NEOP:tr|A0A067R416|A0A067R416_ZOONE:tr|A0A2J7QYF0|A0A2J7QYF0_9NEOP |
| AAAPAVVAAPAVAK | 41.42 | 1205.7131 | 14 | 1 | 1206.7216 | 22.56 | 1.69E+09 | tr|A0A2J7PG65|A0A2J7PG65_9NEOP:tr|A0A2P8XG07|A0A2P8XG07_BLAGE |
| GLGLGHGLGY | 29.87 | 942.4923 | 10 | 0.1 | 943.4996 | 34.29 | 1.62E+09 | tr|A0A2P8YW25|A0A2P8YW25_BLAGE |
| AYDIQDALTGDSK | 37.99 | 1395.6517 | 13 | 2.7 | 1396.6628 | 39.38 | 1.55E+09 | tr|A0A2P8YBK0|A0A2P8YBK0_BLAGE:tr|A0A2P8YBK7|A0A2P8YBK7_BLAGE:tr|A0A2P8YBM8|A0A2P8YBM8_BLAGE:tr|A0A2J7Q400|A0A2J7Q400_9NEOP:tr|A0A2J7Q402|A0A2J7Q402_9NEOP:tr|A0A2P8YBL5|A0A2P8YBL5_BLAGE:tr|A0A2J7R9Q9|A0A2J7R9Q9_9NEOP:tr|A0A067QUK4|A0A067QUK4_ZOONE:tr|A0A2J7Q405|A0A2J7Q405_9NEOP |
| AAVLPVESSVVK | 37.22 | 1197.6969 | 12 | 1 | 1198.7053 | 32.75 | 1.55E+09 | tr|R4V1V4|R4V1V4_COPFO:tr|A0A2J7PEY9|A0A2J7PEY9_9NEOP |
| AEIGIAMGSGTAVAK | 47.04 | 1374.7177 | 15 | 1.7 | 1375.7273 | 30.63 | 1.54E+09 | tr|A0A2J7R7U7|A0A2J7R7U7_9NEOP:tr|A0A2J7R7V8|A0A2J7R7V8_9NEOP:tr|A0A2J7R7U2|A0A2J7R7U2_9NEOP:tr|A0A2J7R7U1|A0A2J7R7U1_9NEOP:tr|A0A067REG0|A0A067REG0_ZOONE:tr|A0A2P8XTT4|A0A2P8XTT4_BLAGE |
| HQGVM(+15.99)VGMGQK | 39.21 | 1186.5587 | 11 | 0.5 | 1187.5665 | 8.53 | 1.43E+09 | tr|Q3HNA6|Q3HNA6_RETFL:tr|A0A067QL86|A0A067QL86_ZOONE:tr|A0A2J7RHY5|A0A2J7RHY5_9NEOP:tr|A0A2Z5RE76|A0A2Z5RE76_9NEOP:tr|A0A067RMN2|A0A067RMN2_ZOONE:tr|A0A2P8Z2J3|A0A2P8Z2J3_BLAGE:tr|A0A2R4CIH4|A0A2R4CIH4_PERAM:tr|A0A2J7PLV1|A0A2J7PLV1_9NEOP |

| | | | | | | | | |
|---|---|---|---|---|---|---|---|---|
| VAAAPVAY | 19 | 760.4119 | 8 | 0.1 | 761.4193 | 26.04 | 1.35E+09 | tr\|A0A2P8XZ18\|A0A2P8XZ18_BLAGE |
| VAAPVAVAA PVAR | 32.2 | 1190.7135 | 13 | 0.3 | 1191.7212 | 29.25 | 1.34E+09 | tr\|A0A2P8YBM8\|A0A2P8YBM8_BLAGE:tr\|A0A2P8YBL7\|A0A2P8YBL7_BLAGE:tr\|A0A2P8YBP8\|A0A2P8YBP8_BLAGE |
| AYDIQDALT GDAK | 40.88 | 1379.6569 | 13 | 2.1 | 1380.667 | 41.24 | 1.27E+09 | tr\|A0A2P8YBM8\|A0A2P8YBM8_BLAGE:tr\|A0A2P8YBL7\|A0A2P8YBL7_BLAGE:tr\|A0A2P8YBV6\|A0A2P8YBV6_BLAGE:tr\|A0A2P8YBK9\|A0A2P8YBK9_BLAGE:tr\|A0A2J7Q3Z2\|A0A2J7Q3Z2_9NEOP:tr\|A0A067QWG3\|A0A067QWG3_ZOONE |
| AYDIQDAITG DAK | 40.88 | 1379.6569 | 13 | 2.1 | 1380.667 | 41.24 | 1.27E+09 | tr\|A0A2P8YBK0\|A0A2P8YBK0_BLAGE:tr\|A0A2P8YBK7\|A0A2P8YBK7_BLAGE |
| VEIDRFPY | 22.06 | 1037.5182 | 8 | 1.9 | 519.7673 | 40.03 | 1.27E+09 | tr\|A0A2J7RQU2\|A0A2J7RQU2_9NEOP:tr\|A0A2P8ZNR8\|A0A2P8ZNR8_BLAGE:tr\|D0VNY6\|D0VNY6_BLAGE:tr\|A0A140KPR2\|A0A140KPR2_BLAGE:tr\|D0VNY7\|D0VNY7_BLAGE |
| VELDRFPY | 22.06 | 1037.5182 | 8 | 1.9 | 519.7673 | 40.03 | 1.27E+09 | tr\|A0A2P0XIG9\|A0A2P0XIG9_PERAM:Q17127\|HEXA_BLADI:tr\|I3TBB2\|I3TBB2_CRYPU |
| KYPDDKPLGF PFD | 33.27 | 1537.7452 | 13 | 1.4 | 769.881 | 50.58 | 1.24E+09 | tr\|A0A2J7RQU2\|A0A2J7RQU2_9NEOP:tr\|Q94643\|Q94643_PERAM:tr\|A0A067RPM2\|A0A067RPM2_ZOONE:tr\|Q25639\|Q25639_PERAM:tr\|A0A0A0QKL4\|A0A0A0QKL4_COPFO |
| DLTDYLMK | 26.37 | 997.479 | 8 | 0.9 | 998.4872 | 52.51 | 1.24E+09 | tr\|Q3HNA6\|Q3HNA6_RETFL:tr\|A0A067QL86\|A0A067QL86_ZOONE:tr\|A0A2J7RHY5\|A0A2J7RHY5_9NEOP:tr\|A0A2Z5RE76\|A0A2Z5RE76_9NEOP:tr\|A0A067RMN2\|A0A067RMN2_ZOONE:tr\|A0A2P8Z2J3\|A0A2P8Z2J3_BLAGE:tr\|A0A2R4CIH4\|A0A2R4CIH4_PERAM:tr\|A0A2J7PLV1\|A0A2J7PLV1_9NEOP:tr\|A0A067R416\|A0A067R416_ZOONE:tr\|A0A2J7QYF0\|A0A2J7QYF0_9NEOP |
| APSFDSAIIK | 34.27 | 1047.5601 | 10 | 1 | 1048.5684 | 31.25 | 1.22E+09 | tr\|A0A2J7PVU6\|A0A2J7PVU6_9NEOP |
| GVVIGTGL | 19.45 | 714.4276 | 8 | 1.2 | 715.4357 | 42.88 | 1.21E+09 | tr\|A0A2J7R7U7\|A0A2J7R7U7_9NEOP:tr\|A0A2J7R7V8\|A0A2J7R7V8_9NEOP:tr\|A0A2J7R7U2\|A0A2J7R7U2_9NEOP:tr\|A0A2J7R7U1\|A0A2J7R7U1_9NEOP:tr\|A0A067REG0\|A0A067REG0_ZOONE:tr\|A0A2P8XTT4\|A0A2P8XTT4_BLAGE |
| YC(+57.02)GYP ENLLLPK | 33.52 | 1465.7275 | 12 | 2.4 | 1466.7383 | 51 | 1.15E+09 | tr\|Q94643\|Q94643_PERAM |
| YC(+57.02)GYP ENLLIPK | 33.52 | 1465.7275 | 12 | 2.4 | 1466.7383 | 51 | 1.15E+09 | tr\|D3JUE9\|D3JUE9_PERAM |
| AAPAVSY | 20.84 | 677.3384 | 7 | 0.3 | 678.3459 | 15.55 | 1.08E+09 | tr\|A0A2P8Z7V6\|A0A2P8Z7V6_BLAGE:tr\|A0A2P8XJ38\|A0A2P8XJ38_BLAGE |

| | | | | | | | | |
|---|---|---|---|---|---|---|---|---|
| MVSDIANAWK | 37.33 | 1133.554 | 10 | -0.8 | 1134.5603 | 34.76 | 1.05E+09 | tr|A0A2J7RAV9|A0A2J7RAV9_9NEOP |
| GAPGFHSPY | 25.86 | 931.4188 | 9 | 0.2 | 932.4263 | 16.65 | 1.05E+09 | tr|A0A067R1R9|A0A067R1R9_ZOONE |
| GLGHGLGY | 19.35 | 772.3868 | 8 | -0.8 | 773.3934 | 15.5 | 1.05E+09 | tr|A0A2J7PG65|A0A2J7PG65_9NEOP:tr|A0A2P8YW25|A0A2P8YW25_BLAGE |
| AVDSLVPIGR | 26 | 1025.5869 | 10 | 1.5 | 513.8015 | 35.26 | 1.03E+09 | tr|A0A2P8ZHF8|A0A2P8ZHF8_BLAGE:tr|A0A2J7Q0B3|A0A2J7Q0B3_9NEOP |
| GLGSGLGY | 16.5 | 722.3599 | 8 | 0.3 | 723.3674 | 30.1 | 1.03E+09 | tr|A0A2P8YW25|A0A2P8YW25_BLAGE |
| VDIATPVVK | 24.25 | 940.5593 | 9 | -0.5 | 941.5662 | 23.61 | 1.01E+09 | tr|A0A2P8YNR5|A0A2P8YNR5_BLAGE:tr|A0A067RB44|A0A067RB44_ZOONE |
| TVPIYEGY | 17.47 | 940.4542 | 8 | 1.5 | 941.4629 | 40.91 | 9.93E+08 | tr|Q3HNA6|Q3HNA6_RETFL:tr|A0A067QL86|A0A067QL86_ZOONE:tr|A0A2J7RHY5|A0A2J7RHY5_9NEOP:tr|A0A2Z5RE76|A0A2Z5RE76_9NEOP:tr|A0A067RMN2|A0A067RMN2_ZOONE:tr|A0A2P8Z2J3|A0A2P8Z2J3_BLAGE:tr|A0A2R4CIH4|A0A2R4CIH4_PERAM:tr|A0A2J7PLV1|A0A2J7PLV1_9NEOP:tr|A0A067R416|A0A067R416_ZOONE:tr|A0A2J7QYF0|A0A2J7QYF0_9NEOP |
| DLLDQIGEGGR | 29.06 | 1171.5833 | 11 | 2 | 1172.5929 | 46.51 | 9.78E+08 | tr|A0A2J7QRJ1|A0A2J7QRJ1_9NEOP:tr|A0A2P9A976|A0A2P9A976_BLAGE |
| NEVPPHIF | 24.94 | 951.4814 | 8 | 0.1 | 952.4888 | 31.85 | 9.45E+08 | tr|A0A2J7QRJ1|A0A2J7QRJ1_9NEOP:tr|A0A2P9A976|A0A2P9A976_BLAGE:tr|A0A2P8XQ42|A0A2P8XQ42_BLAGE |
| FVEWIPNNVK | 29.19 | 1244.6553 | 10 | 1.3 | 1245.6642 | 46.45 | 9.31E+08 | tr|A0A2J7QIE5|A0A2J7QIE5_9NEOP:tr|A0A067R568|A0A067R568_ZOONE:tr|A0A2J7QSV2|A0A2J7QSV2_9NEOP:tr|A0A2J7QRF5|A0A2J7QRF5_9NEOP:tr|A0A2J7PKK2|A0A2J7PKK2_9NEOP:tr|A0A067QYS6|A0A067QYS6_ZOONE:tr|A0A2J7R1W4|A0A2J7R1W4_9NEOP:tr|A1C231|A1C231_CRYPU:tr|A0A2P8YDV3|A0A2P8YDV3_BLAGE |
| AGLQFPVGR | 23.4 | 943.5239 | 9 | 0.6 | 472.7695 | 30.42 | 9.28E+08 | tr|A0A2J7RT92|A0A2J7RT92_9NEOP:tr|A0A067QGN4|A0A067QGN4_ZOONE:tr|A0A067QYW8|A0A067QYW8_ZOONE:tr|A0A067RLA1|A0A067RLA1_ZOONE:tr|A0A2P8XJ80|A0A2P8XJ80_BLAGE:tr|A0A067RTR6|A0A067RTR6_ZOONE:tr|A0A2J7RNR6|A0A2J7RNR6_9NEOP:tr|A0A126QCW4|A0A126QCW4_9NEOP:tr|A0A140D4D2|A0A140D4D2_9NEOP:tr|A0A223FVZ4|A0A223FVZ4_9NEOP:tr|A0A2P8XJ03|A0A2P8XJ03_BLAGE:tr|A0A067R0G4|A0A067R0G4_ZOONE:tr|R4UK09|R4UK09_COPFO |
| LIDFGLATK | 26.22 | 976.5593 | 9 | 0.8 | 977.5674 | 45.24 | 8.99E+08 | tr|A0A2P8YXL7|A0A2P8YXL7_BLAGE:tr|A0A2J7PNB1|A0A2J7PNB1_9NEOP:tr|A0A2J7PNB7|A0A2J7PNB7_9NEOP |

| | | | | | | | | |
|---|---|---|---|---|---|---|---|---|
| ISLTTQLEDTKR | 28.81 | 1403.762 | 12 | -0.3 | 702.8881 | 19.96 | 8.95E+08 | tr\|A0A2J7QRJ1\|A0A2J7QRJ1_9NEOP:tr\|A0A2P9A976\|A0A2P9A976_BLAGE:tr\|A0A2P8XQ42\|A0A2P8XQ42_BLAGE |
| KYPDDKPLGFPF | 33.78 | 1422.7183 | 12 | 0.9 | 712.3671 | 50.81 | 8.76E+08 | tr\|A0A2J7RQU2\|A0A2J7RQU2_9NEOP:tr\|Q94643\|Q94643_PERAM:tr\|A0A067RPM2\|A0A067RPM2_ZOONE:tr\|Q25639\|Q25639_PERAM:tr\|A0A0A0QKL4\|A0A0A0QKL4_COPFO |
| ITLELGGK | 24.14 | 829.4909 | 8 | 1.3 | 830.4993 | 23.29 | 8.51E+08 | tr\|A0A2J7PWN9\|A0A2J7PWN9_9NEOP:tr\|A0A2P0XIF5\|A0A2P0XIF5_PERAM:tr\|A0A2H4V6Z3\|A0A2H4V6Z3_MASDA |
| ITIEIGGK | 24.14 | 829.4909 | 8 | 1.3 | 830.4993 | 23.29 | 8.51E+08 | |
| AAPAVAAHGLLGVAY | 35.67 | 1379.7561 | 15 | 0.2 | 690.8855 | 51.36 | 8.41E+08 | tr\|A0A2P8XZ18\|A0A2P8XZ18_BLAGE |
| SLVEPDGTR | 19.24 | 972.4876 | 9 | -0.4 | 973.4945 | 10.51 | 8.40E+08 | tr\|A0A2P8YBK0\|A0A2P8YBK0_BLAGE:tr\|A0A2P8YBK7\|A0A2P8YBK7_BLAGE:tr\|A0A2P8YBM8\|A0A2P8YBM8_BLAGE:tr\|A0A2J7Q400\|A0A2J7Q400_9NEOP:tr\|A0A2J7Q402\|A0A2J7Q402_9NEOP:tr\|A0A2P8YBL5\|A0A2P8YBL5_BLAGE:tr\|A0A2J7Q407\|A0A2J7Q407_9NEOP:tr\|A0A2J7Q404\|A0A2J7Q404_9NEOP:tr\|A0A2J7Q405\|A0A2J7Q405_9NEOP:tr\|Q5H7A6\|Q5H7A6_9NEOP:tr\|A0A2J7Q412\|A0A2J7Q412_9NEOP:tr\|A0A067QUK2\|A0A067QUK2_ZOONE:tr\|A0A2P8Y146\|A0A2P8Y146_BLAGE:tr\|A0A2P8Y131\|A0A2P8Y131_BLAGE |

**Table S4.** Identified potential biomarkers in serum regulated by APE

| Proposed identity | Formula | Rt (min) | Theoretical (m/z) | Experimental (m/z) | Error (ppm) | VIP | P(corr) | MS/MS | Ion Mode | Change Trend (M/C) | Change Trend (M+HAPE/M) |
|---|---|---|---|---|---|---|---|---|---|---|---|
| Hydroxyeicosate-traenoate | $C_{20}H_{32}O_3$ | 1.34 | 319.22751 | 319.22751 | 0.167 | 5.70481 | -0.669338 | 319(49), 203(23), 301(43), 115(100) | N | ↑* | ↓ |
| (S)-Ureidoglycolate | $C_3H_6N_2O_4$ | 1.52 | 133.02419 | 133.0242 | -1.377 | 1.82445 | 0.496272 | 133(100), 112(1), 95(13), 80(1) | N | ↓ | ↑# |
| Tryptamine | $C_{10}H_{12}N_2$ | 1.54 | 161.10691 | 161.10689 | -2.700 | 1.50064 | 0.430885 | 161(100), 142(24), 131(5), 116(100), 73(25) | P | ↓* | ↑# |
| Uric acid | $C_5H_4N_4O_3$ | 1.82 | 167.02106 | 167.02098 | -2.287 | 1.98642 | 0.458843 | 167(100), 147(4), 124(47), 96(7) | N | ↓* | ↑### |
| 2,3-Dihydroxyindole | $C_8H_7NO_2$ | 2.17 | 150.05482 | 150.0547 | -1.700 | 1.95097 | 0.655555 | 150(10), 133(61), 104(100), 102(26) | P | ↓ | ↑ |
| Glycerone | $C_3H_6O_3$ | 2.2 | 89.02307 | 89.02301 | -3.488 | 2.00319 | 0.422191 | 89(100), 71(5) | N | ↓ | ↑ |
| Acetone cyanohydrin | $C_4H_7NO$ | 2.24 | 86.06037 | 86.06044 | 4.643 | 1.55296 | 0.522295 | 86(91), 71(2), 69(100), 57(31) | P | ↑ | ↓# |
| Nicotinamide | $C_6H_6N_2O$ | 2.27 | 123.0553 | 123.05527 | -0.158 | 1.92451 | 0.692903 | 123(13), 118(70), 100(100), 95(17) | P | ↑* | ↓ |
| Methyl-2-pyrrolidinone | $C_5H_9NO$ | 2.53 | 100.07587 | 100.07594 | 2.493 | 1.80280 | 0.63572 | 100(70), 89(13), 72(97), 55(100) | P | ↑* | ↓ |
| Hypoxanthine | $C_5H_4N_4O$ | 3.48 | 135.03006 | 135.03000 | -1.017 | 2.74366 | -0.45807 | 135(100), 122(1), 99(3) | N | ↑** | ↓### |
| Methyl-2-oxopentanoate | $C_6H_{10}O_3$ | 3.94 | 129.05444 | 129.05441 | -1.632 | 8.41826 | -0.676791 | 129(100), 117(49), 105(46), 100(52) | N | ↑ | ↓# |
| Adenine | $C_5H_5N_5$ | 4.04 | 134.04602 | 134.04599 | -0.983 | 4.00772 | 0.572833 | 134(40), 120(15), 75(5) | N | ↑*** | ↓### |
| Adenosine | $C_{10}H_{13}N_5O_4$ | 4.08 | 268.10338 | 268.10352 | -1.904 | 1.83707 | 0.593624 | 268(100), 213(15), 199(11), 159(10), 95(25) | P | ↑*** | ↓## |
| gamma-Glutamyl-beta-cyanoalanine | $C_9H_{13}N_3O_5$ | 5.60 | 244.09231 | 244.09241 | -0.382 | 1.62911 | 0.558153 | 244(100), 226(2), 180(18), 85(1) | P | ↑** | ↓### |
| Cytosine | $C_4H_5N_3O$ | 6.69 | 112.05064 | 112.05067 | 1.175 | 1.99926 | 0.639902 | 112(100), 95(12), 70(21) | P | ↓** | ↑ |
| Arginine | $C_6H_{14}N_4O_2$ | 6.75 | 174.20114 | 174.20115 | 1.011 | 3.10672 | 0.451013 | 174(100), 159(5), 132(17), 118(6) | P | ↓* | ↑### |
| Glycocholate | $C_{26}H_{43}NO_6$ | 7.43 | 464.30101 | 464.30121 | 1.175 | 3.39934 | -0.458653 | 464(100), 430(2), 330(2), 74(32) | N | ↑** | ↓# |
| Trimethylamine N-oxide | $C_3H_9NO$ | 8.18 | 76.07625 | 76.07623 | 3.092 | 1.57888 | 0.480898 | 76(100), 59(78) | P | ↓* | ↑ |
| O-Propanoylcarnitine | $C_{10}H_{19}NO_4$ | 8.34 | 218.13835 | 218.13846 | -1.030 | 1.64078 | 0.548771 | 218(36), 159(15), 85(100), 60(13) | P | ↑* | ↓# |

| | | | | | | | | | | | |
|---|---|---|---|---|---|---|---|---|---|---|---|
| N-Acetylserotonin | $C_{12}H_{14}N_2O_2$ | 8.48 | 219.11608 | 219.11592 | 4.220 | 1.72386 | 0.614361 | 219(42), 202(31),184(24), 124(11), 116(16) | P | ↑* | ↓# |
| Proline | $C_5H_9NO_2$ | 8.62 | 116.07054 | 116.07061 | -0.042 | 3.0198 | 0.476260 | 116(36), 92(42), 74(100), 57(5) | P | ↑* | ↓# |
| Phenindamine | $C_{19}H_{19}N$ | 8.67 | 262.1638 | 262.16458 | -1.198 | 1.58702 | 0.513712 | 262(100), 244(4), 200(42), 88(10) | P | ↑* | ↓ |
| 3-Methyleneoxindole | $C_9H_7NO$ | 8.91 | 146.05974 | 146.05981 | -1.578 | 1.53085 | 0.432814 | 146(61), 127(3), 109(60), 82(100) | P | ↑ | ↓# |
| 3-Indoleacrylate | $C_{11}H_9NO_2$ | 8.95 | 188.07029 | 188.07036 | -1.303 | 1.62697 | 0.505108 | 188(33), 170(7), 146(100), 118(25) | P | ↓* | ↑# |
| trans-Cinnamate | $C_9H_8O_2$ | 9.00 | 147.04406 | 147.04395 | -0.721 | 2.23304 | 0.698291 | 147(2), 123(7), 102(100), 58(40) | N | ↑ | ↓ |
| Phenylalanine | $C_9H_{11}NO_2$ | 9.01 | 164.07055 | 164.07053 | -0.458 | 7.06554 | 0.486796 | 164(10), 147(100), 136(9), 96(12), 72(34) | N | ↓* | ↑## |
| 2-Phenylacetamide | $C_8H_9NO$ | 9.02 | 136.0754 | 136.07553 | -1.179 | 1.5497 | -0.504565 | 136(100), 119(6), 109(3), 91(15) | P | ↓** | ↑ |
| Taurocholic acid | $C_{26}H_{45}NO_7S$ | 9.35 | 514.28365 | 514.28381 | 0.992 | 2.58934 | -0.421297 | 514(100), 496(1), 124(13), 106(1) | N | ↓* | ↑ |
| Valine | $C_5H_{11}NO_2$ | 9.35 | 118.08626 | 118.08622 | -1.108 | 2.8162 | -0.53002 | 118(60), 76(22), 72(100), 59(11) | P | ↓* | ↑# |
| Threonine | $C_4H_9NO_3$ | 9.39 | 120.06551 | 120.06561 | 0.752 | 1.79882 | 0.530487 | 120(22), 78(10), 74(100), 56(63) | P | ↓* | ↑# |
| Pipecolate | $C_6H_{11}NO_2$ | 9.45 | 130.08589 | 130.08621 | -0.158 | 1.80951 | 0.598083 | 130(23), 84(100), 56(1) | P | ↑* | ↓ |
| 3-Sulfino-L-alanine | $C_3H_7NO_4S$ | 9.5 | 152.00119 | 152.00117 | -0.229 | 4.49157 | 0.440294 | 152(3), 108(5), 92(100), 80(6) | N | ↓* | ↑# |
| Glutamate | $C_5H_8O_4N$ | 9.68 | 146.04462 | 146.04488 | 1.601 | 2.13824 | -0.45007 | 146(10), 123(8), 103(15), 61(100) | N | ↓* | ↑## |
| (E)-4-Trimethylammoniot-2-enoate | $C_7H_{13}NO_2$ | 10.27 | 144.10163 | 144.10176 | -1.008 | 1.83857 | 0.564788 | 144(100), 117(10), 85(4) | P | ↑* | ↓ |
| Tryptophan | $C_{11}H_{12}N_2O_2$ | 8.94 | 205.09475 | 205.09683 | -0.324 | 2.01713 | -0.689973 | 205(2), 188(100), 146(69), 132(6) | P | ↓** | ↑## |
| Indol | $C_8H_7N$ | 9.08 | 118.08626 | 118.06547 | 0.344 | 1.97085 | 0.612589 | 118(32), 103(42), 87(100) | P | ↓** | ↑# |

Delta: system error, *$p$<0.05,**$p$<0.01, ***$p$<0.001, M group vs Con group, #$p$<0.05, ##$p$<0.01, ###$p$<0.001, M group vs M+HAPE group

**Table S5.** Identified potential biomarkers in faeces regulated by APE

| Proposed identity | Formula | Rt (min) | Theoretical (m/z) | Experimental (m/z) | RDB (ppm) | VIP | P(corr) | MS/MS | Ion Mode | Chang e Trend (M/C) | Change Trend (M+HA PE/M) |
|---|---|---|---|---|---|---|---|---|---|---|---|
| Thymine | $C_5H_6O_2N_2$ | 1.52 | 125.03456 | 125.03444 | -0.911 | 3.00458 | -0.53094 | 125(100), 72(1), 55(1) | N | ↑* | ↓# |
| Tryptamine | $C_{10}H_{12}N_2$ | 1.54 | 161.10691 | 161.10689 | -2.700 | 1.50064 | 0.430885 | 205(2), 188(100), 146(69), 132(6) | P | ↑** | ↓# |
| 7,8-dihydroxykynurenate | $C_{10}H_9NO_5$ | 1.55 | 222.04017 | 222.04024 | 2.437 | 5.00473 | 0.476098 | 222(14), 160(100), 134(17), 91(3) | N | ↑ | ↓# |
| Uracil | $C_4H_4O_2N_2$ | 1.82 | 111.01868 | 111.01873 | -1.566 | 4.00817 | -0.477916 | 111(100), 83(2), 78(71) | N | ↑* | ↓ |
| Hippurate | $C_9H_9NO_3$ | 1.92 | 178.04991 | 178.05009 | 1.238 | 4.44243 | 0.609988 | 178(100), 79(11), 55(1) | N | ↓* | ↑# |
| Glycerone | $C_3H_6O_3$ | 2.23 | 89.02301 | 89.0231 | -2.477 | 2.48627 | -0.484086 | 89(100), 75(25), 59(1) | N | ↓* | ↑ |
| Xanthine | $C_5H_4N_4O_2$ | 2.45 | 153.04033 | 153.04033 | -2.430 | 1.84488 | -0.467949 | 153(38), 109(100), 108(30), 85(2) | P | ↑* | ↓ |
| Nicotinate | $C_6H_5NO_2$ | 2.46 | 124.03917 | 124.03915 | -1.249 | 2.28845 | -0.416789 | 124(100), 112(2), 104(6), 80(8) | P | ↑ | ↓## |
| N-Methyl-2-pyrrolidinon e | $C_5H_9NO$ | 2.54 | 100.07585 | 100.07581 | 1.194 | 2.64073 | 0.588156 | 100(100), 85(1), 72(2), 56(15) | P | ↓ | ↑## |
| Pyridoxine | $C_8H_{11}NO_3$ | 2.83 | 170.0808 | 170.08073 | -2.586 | 2.90206 | 0.575481 | 170(100), 152(1), 128(1) | P | ↑* | ↓# |
| N,N-Dihydroxy-L-isoleuc ine | $C_6H_{13}NO_4$ | 2.87 | 164.09261 | 164.09273 | -2.084 | 2.01201 | 0.402505 | 164(100), 146(1), 122(8), 95(1) | P | ↓* | ↑ |
| Piperidine | $C_5H_{11}N$ | 3.21 | 86.09686 | 86.09679 | 4.228 | 2.67982 | 0.535767 | 86(100), 72(22), 58(9) | P | ↓** | ↑ |
| Hypoxanthine | $C_5H_4ON_4$ | 3.42 | 135.03002 | 135.03011 | -0.202 | 2.36685 | -0.451332 | 135(100), 99(1), 87(1), 65(6) | N | ↓* | ↑ |
| N-Methyl-2-pyridone-5-carboxamide | $C_7H_8N_2O_2$ | 3.60 | 153.06542 | 153.06543 | -2.771 | 2.71579 | 0.557247 | 153(100), 136(4), 128(4), | P | ↓* | ↑ |

| | | | | | | | | | | | |
|---|---|---|---|---|---|---|---|---|---|---|---|
| | | | | | | | | 110(25) | | | |
| Tazarotene | $C_{21}H_{21}NO_2S$ | 3.95 | 350.1456 | 350.1456 | 2.469 | 2.56927 | -0.491255 | 350(5), 170(15), 98(11), 87(100) | N | ↑ | ↓# |
| 3-Methyladenine | $C_6H_7N_5$ | 4.84 | 150.07709 | 150.0771 | -2.144 | 4.00493 | -0.668376 | 150(11), 133(61), 104(100), 87(10) | P | ↑* | ↓ |
| Cytosine | $C_4H_5N_3O$ | 4.85 | 112.05053 | 112.05055 | 0.104 | 2.27972 | 0.60472 | 112(100), 95(10), 88(27), 70(93) | P | ↓ | ↑# |
| 4-Pyridoxate | $C_8H_9NO_4$ | 5.84 | 182.04494 | 182.04507 | 1.570 | 3.8611 | -0.429172 | 182(27), 138(100), 120(2), 108(27) | N | ↑* | ↓# |
| Guanine | $C_5H_5N_5O$ | 5.90 | 152.05621 | 152.05638 | 1.069 | 4.35983 | 0.546908 | 152(100), 136(4), 128(4), 110(36) | P | ↓ | ↑# |
| N-Acetylisatin | $C_{10}H_7NO_3$ | 8.33 | 188.03457 | 188.03455 | 1.757 | 7.49959 | 0.61269 | 188(3), 144(100), 116(3), 92(1) | N | ↑ | ↓# |
| N-Acetyl-L-lysine | $C_8H_{16}N_2O_3$ | 8.44 | 189.12311 | 189.12299 | -2.004 | 1.91799 | 0.524277 | 189(4), 143(36), 132(67), 86(100) | P | ↓* | ↑ |
| Propranolol | $C_{16}H_{21}NO_2$ | 8.53 | 258.1457 | 258.14594 | -1.023 | 1.94572 | 0.432626 | 258(91), 240(67), 214(10), 196(47) | N | ↑* | ↓ |
| Proline | $C_5H_9NO_2$ | 8.62 | 116.07059 | 116.0706 | -0.044 | 3.24898 | 0.476781 | 116(17), 92(5), 70(100) | P | ↑* | ↓### |
| Oxadixyl | $C_{14}H_{18}N_2O_4$ | 8.98 | 279.13292 | 279.13315 | 2.003 | 2.03144 | -0.464018 | 279(31), 136(99), 116(100), 70(16) | P | ↑ | ↓ |
| Tolylacetonitrile | $C_9H_9N$ | 9.01 | 132.08045 | 132.08054 | -1.786 | 6.72476 | -0.512432 | 132(2), 86(100), 69(2) | P | ↓* | ↑ |
| Tryptophan | $C_{11}H_{12}N_2O_2$ | 9.02 | 205.09667 | 205.09668 | -2.312 | 7.31568 | 0.418562 | 205(2), 188(100), 146(73), 144(13) | P | ↓* | ↑## |
| trans-Cinnamate | $C_9H_8O_2$ | 9.11 | 147.04386 | 147.04408 | 0.163 | 1.51218 | -0.739326 | 147(3), 102(19), 87(11), 69(14), 61(100) | N | ↑* | ↓ |
| Phenylalanine | $C_9H_{11}NO_2$ | 9.11 | 166.08582 | 166.08582 | -2.620 | 2.18964 | -0.407504 | 166(6), 142(4), 120(100), | P | ↑* | ↓## |

| | | | | | | | | | | | |
|---|---|---|---|---|---|---|---|---|---|---|---|
| | | | | | | | | 103(4) | | | |
| Indole | $C_8H_7N$ | 9.13 | 118.06502 | 118.06508 | -0.388 | 2.64653 | 0.409402 | 118(17), 105(3), 95(6), 72(100) | P | ↓* | ↑## |
| 3-Methylene-indolenine | $C_9H_7ON$ | 9.17 | 144.04424 | 144.04443 | 0.275 | 2.71243 | 0.417228 | 144(100), 116(2), 69(4) | N | ↓* | ↑## |
| 4-Hydroxy-2-quinolinecarboxylic acid | $C_{10}H_7NO_3$ | 9.18 | 188.03443 | 188.03452 | 1.598 | 3.6812 | 0.429091 | 188(100), 146(23), 132(16), 92(7) | N | ↓ | ↑## |
| Valine | $C_5H_{11}NO_2$ | 9.35 | 118.08623 | 118.08622 | -0.298 | 3.84014 | -0.566811 | 118(100), 146(24), 132(16) | P | ↑* | ↓### |
| Serine | $C_3H_7O_3N$ | 9.63 | 104.03403 | 104.03408 | -1.342 | 1.78605 | -0.598565 | 104(2), 90(100), 71(32) | N | ↓* | ↑# |
| Glutamate | $C_5H_8O_4N$ | 9.64 | 146.04469 | 146.04488 | 0.656 | 4.12435 | -0.473216 | 146(69), 132(26), 87(12), 51(5) | N | ↑* | ↓## |
| 3-Acetamidopropanal | $C_5H_9NO_2$ | 9.79 | 116.07059 | 116.07061 | 0.042 | 5.02436 | -0.605067 | 116(100), 92(25), 70(3) | P | ↓ | ↑ |
| Methylene-L-glutamine | $C_6H_{10}N_2O_3$ | 10.31 | 159.07602 | 159.0761 | -2.004 | 2.64587 | -0.574584 | 159(100), 144(11), 131(18), 67(2) | P | ↓ | ↑## |
| Ornithine | $C_5H_{12}O_2N_2$ | 10.33 | 131.08132 | 131.0815 | -0.032 | 3.12551 | -0.532667 | 131(40), 121(2), 118(2), 85(100) | N | ↑* | ↓# |
| Citrulline | $C_6H_{13}N_3O_3$ | 10.37 | 176.10257 | 176.1026 | -2.089 | 2.32574 | -0.565668 | 176(39), 159(54), 140(18), 130(31), 87(100) | P | ↑* | ↓## |

Delta: system error, *$p$<0.05,**$p$<0.01, ***$p$<0.001, M group vs Con group, #$p$<0.05, ##$p$<0.01, ###$p$<0.001, M group vs M+HAPE group.

**Table S6.** Linear regression equation and quantitative limits of amino acids

| Name | RT (min) | Linear equation | Correlation coefficient (r) | Linearity range (ng/mL) | Limit of quantificatio n (ng/mL) |
|---|---|---|---|---|---|
| Alanine | 4.23 | y=0.01459 x + 0.02578 | 0.99825 | 5-1250 | 5 |
| Proline | 4.47 | y=0.11913 x + 0.03634 | 0.99421 | 0.5-125 | 0.5 |
| Valine | 4.88 | y=0.10965 x + 0.05735 | 0.99681 | 0.5-125 | 0.5 |
| Threonine | 4.29 | y=0.01475 x + 0.11546 | 0.99572 | 20-5000 | 20 |
| Glutamate | 4.37 | y=0.05369 x+ 0.05723 | 0.99036 | 1-250 | 1 |
| Phenylalanine | 9.31 | y=0.13259 x + 0.03824 | 0.99769 | 0.5-125 | 0.5 |
| Arginene | 3.77 | y=0.02876 x + 0.00305 | 0.99573 | 1-250 | 1 |
| Tryptophan | 11.19 | y=0.15131 x + 0.00966 | 0.99983 | 0.5-62.5 | 0.5 |

**Table S7.** Stability of various targeting substances in QC samples

| Name | RSD (%) | Name | RSD (%) |
|---|---|---|---|
| Alanine | 1.6229 | Glutamate | 1.8797 |
| Proline | 2.5268 | Phenylalanine | 1.8193 |
| Valine | 1.5226 | Arginene | 1.4680 |
| Threonine | 2.7277 | Tryptophan | 2.8011 |

**Table S8.** Quantitative ion pairs of amino acids

| **name** | **Parent ion** | **daughter ion** | **DP** | **EP** | **CE** | **CXP** |
|---|---|---|---|---|---|---|
| Alanine | 90.1 | 44.1 | 40 | 10 | 19 | 5 |
| Proline | 116 | 70 | 40 | 10 | 20 | 8 |
| Valine | 118 | 72 | 14 | 10 | 15 | 8 |
| Threonine | 120.1 | 102.1 | 15 | 10 | 11 | 11 |
| Glutamine | 148.1 | 84.1 | 20 | 10 | 21 | 10 |
| Phenylalanine | 166.1 | 120.2 | 40 | 10 | 18 | 14 |
| Arginine | 175.1 | 70 | 33 | 10 | 26 | 8 |
| Tryptophan | 205.1 | 187.9 | 40 | 10 | 14 | 10 |
| Tryptophan-2,3,3,-d3 | 208.2 | 191 | 40 | 10 | 14 | 21 |

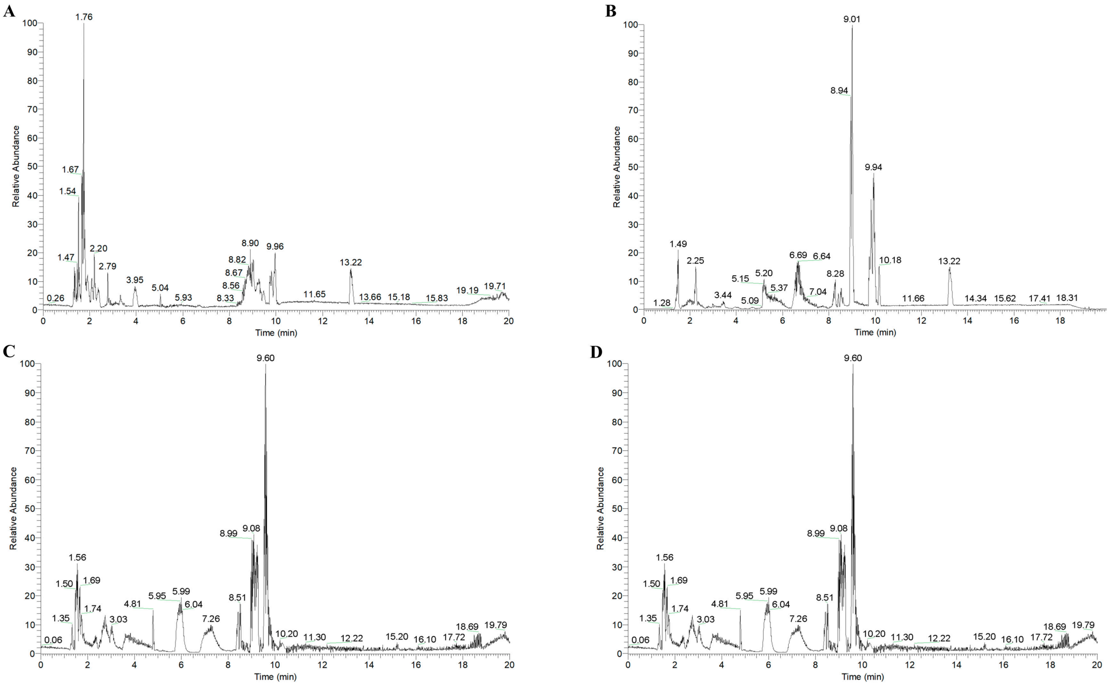

**Figure S1.** The Base Peak plots of QC samples in the positive and negative ions for serum and faeces samples. (**A**) The Base Peak plot of QC sample of serum samples in negative ion mode; (**B**) The Base Peak plot of QC sample of serum samples in positive ion mode; (**C**) The Base Peak plot of QC sample of faeces samples in negative ion mode; (**D**) The Base Peak plot of QC sample of faeces samples in positive ion mode.

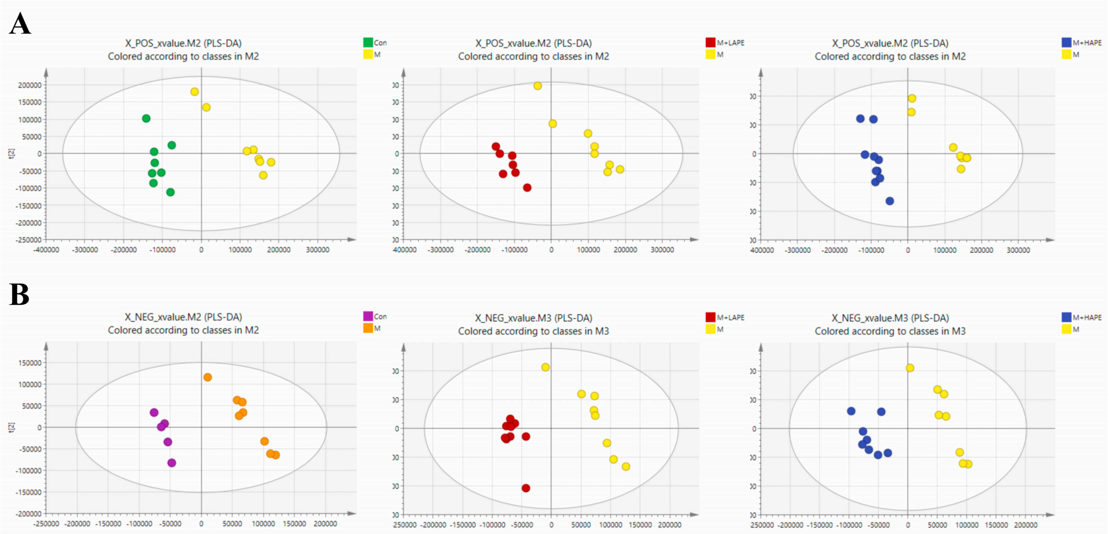

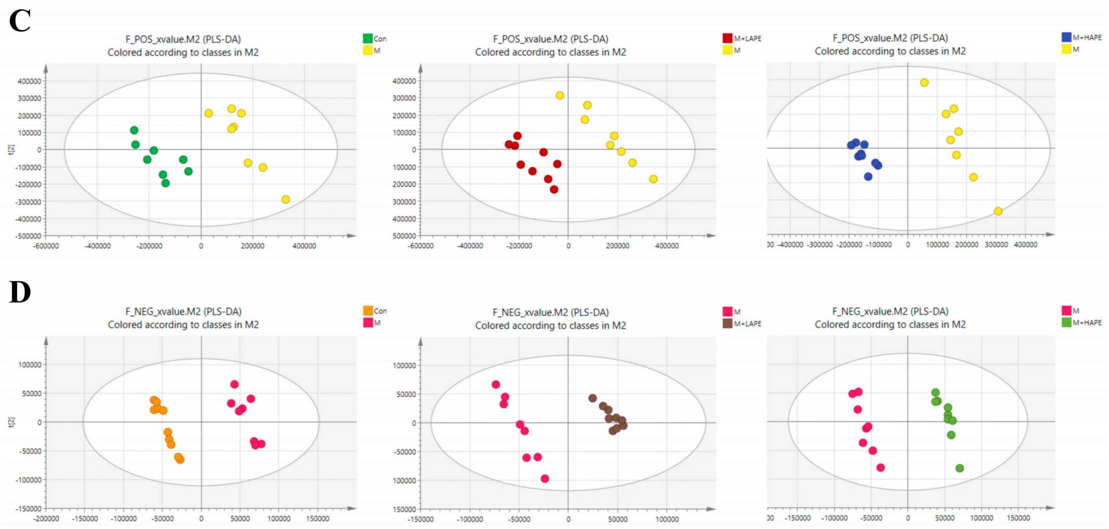


**Figure S2.** The result of PLS-DA analysis based on SCIMA_P 14.0. (**A**) The PLS-DA analysis of serum samples in positive ion mode; (**B**) The PLS-DA analysis of serum samples in negative ion mode; (**C**) The PLS-DA analysis of faeces samples in positive ion mode; (**D**) The PLS-DA analysis of faeces samples in negative ion mode. Con: control group, M: model group, M+LAPE: the low dose group of APE, M+HAPE: the high dose group of APE.
